# Food reward entrainment increases mealtime anxiety in goldfish via a ghrelin-dependent mechanism

**DOI:** 10.1038/s41598-025-13194-x

**Published:** 2025-07-30

**Authors:** Lisbeth Herrera-Castillo, Nuria Saiz, Nuria de Pedro, Esther Isorna

**Affiliations:** Department of Genetics, Physiology and Microbiology, Faculty of Biological Sciences, Complutense University of Madrid, C/Jose Antonio Nováis, Nº12, 28040 Madrid, Spain

**Keywords:** Feeding entrained oscillator (FEO), Teleosts, Food anticipatory activity (FAA), Food seeking, Ghrelin, Hedonic system, Neuroscience, Circadian rhythms and sleep, Feeding behaviour, Motivation, Reward

## Abstract

Food anticipatory activity (FAA), the increase in locomotor activity prior to a scheduled feeding time, is linked not only to energy demands but also to food reward expectations. However, the mechanisms behind FAA remain unclear. We hypothesize that FAA induces anxiety due to reward anticipation. To test this, anxiety-like behavior was assessed in goldfish using open field and black-and-white preference tests under different feeding conditions and daytimes. Increased thigmotactic and scototactic behavior during FAA, compared to the post-FAA period, was observed, even in animals under constant light. Additionally, an unexpected meal did not reduce anxiety, while more hours of fasting did not increase it, suggesting that FAA is an anxiogenic state driven by a food-entrained oscillator rather than energy status. Ghrelin signaling was further explored using two antagonists, both of which reduced anxiety-like behavior when administered during FAA. Moreover, ghrelin administration induced anxiety-like behavior that was reversed by co-injection with an antagonist, supporting its anxiogenic role. These findings suggest a shared neural mechanism between FAA and anxiety, mediated by ghrelin. The possible involvement of ghrelin in activating dopamine-reward circuits in teleosts should be explored in future studies to gain further insight into the neurobiological basis of food anticipatory activity.

**Supplementary Information:**

The online version contains supplementary material available at 10.1038/s41598-025-13194-x.

## Introduction

The circadian system enables organisms to align their behavior and physiological functions with environmental cycles, allowing them to anticipate external changes^1^. This system comprises a network of endogenous oscillators that are synchronized by daily environmental cues known as *Zeitgebers*. The photocycle is the most relevant *Zeitgeber*, and clocks synchronized by it are called LEOs^2^ (light-entrainable oscillators). Endogenous clocks are widely distributed in the organism and function in a similar molecular way in all vertebrates. However, the interaction among the clocks that constitute the circadian system can vary. In mammals, this system is hierarchical, with a master clock located in the suprachiasmatic nucleus (SCN) of the hypothalamus, which functions as a LEO, and it is responsible for synchronizing all other oscillators^1^. In fish, circadian system is less hierarchical as no primary oscillator has been identified so far, with LEOs and FEOs present even in peripheral structures^3^, and rhythmic behavior also being more flexible^4^.

The regulation of food intake in vertebrates is a multidimensional process that involves homeostatic, hedonic and circadian factors^5^. These mechanisms, broadly conserved across vertebrates^6^, collectively determine the quantity, timing, and quality (food type) of ingested nutrients^7^. The homeostatic regulation is the best studied, which responds to orexigenic and satiety signals, and the hedonic system, which links pleasure and reward to eating^6,8^. The involvement of the circadian system on feeding regulation has been less explored. It controls daily rhythms in numerous physiological processes, including feeding behavior, probably driven by circadian rhythms in central areas involved in homeostatic and hedonic feeding regulation^5,9,10^.

One of the most consistent demonstrations of the involvement of the circadian system in feeding behavior is the existence of the Food Anticipatory Activity (FAA), an increment in locomotor activity that animals display prior to food access when food is restricted to a time window and it is not available *ad libitum*^11,12^. FAA persists even in the absence of the light–dark cycle, and it is present in both light and dark phases^13,14^. Due to its importance, the FAA has undergone extensive research, however, the underlying mechanisms that cause this behavior remain elusive. Classical circadian studies generally assume that the FAA is driven by FEOs (food-entrainable oscillators), i.e. clocks entrained by time-restricted feeding schedule. This terminology follows that used for LEOs^3,15^. However, an anatomical structure for this functional FEO has not been found to date, despite numerous studies attempting to identify it^3,16^. Probably because FAA is the result of complex learning, that leads animals to synchronize their physiology and behavior with the expected arrival of food, which involves a network of structures beyond the canonical circadian clocks. In this sense the suprachiasmatic nucleus, which is considered the master clock in mammals, is not necessary for generating FAA^16,17^, and FAA has been also linked with the motivational system that modulates feeding behavior^10,16^. Rodents fed ad libitum with regular chow food, but with a palatable diet offered with a time-restricted feeding schedule showed a FAA prior to expected arrival of the palatable diet (separating metabolic and reward signals that could synchronize the circadian system)^18,19^. Moreover, several hormones and neurotransmitters related with the reward system can modulate FAA, such as ghrelin and orexin^3,16^, dopamine^20–22^, or nuclear receptors such as REV-ERB^23,24^. For this reason, in addition to its classically circadian nature, more recent findings in mammals suggest that FAA might operate through control mechanisms like those identified for reward-seeking behaviors related to addiction^19,25^. Therefore, we hypothesize that during the FAA animals may display signs of anxiety due to their expectation of the reward, which is the arrival of food. In accordance with the aforementioned statements, some feeding regulators and potential mediators of FAA, including orexin and neuropeptide Y, induce anxiety-like behaviors in mammals and fish^26–28^. In addition, daily rhythms in anxiety-like responses have been also described in mammals^29,30^ and fish^31^.

The goldfish (*Carassius auratus*) has been identified as a valuable fish model for studying circadian system and feeding regulation^3,5^. The occurrence of FAA when food is provided on a restricted schedule in this species has been extensively documented^11,32,33^. Although neural mechanisms involved in FAA remain unknown, the involvement of some feeding regulators has been demonstrated in goldfish. Ghrelin (orexigenic) potentiates FAA in goldfish^34^, while the anorexigenic REV-ERBα reduce it^35^. Furthermore, anxiety-like behavior can be measured in this species^27,36^. Considering the previous considerations, the aim of the present study was to investigate whether FAA is an anxiety-like state in teleost, which would be driven by a FEO, using goldfish as a model. For that purpose, we measured anxiety-like responses in fish during their FAA, and 4 h later, after feeding. Moreover, we also compared anxiety-like responses in fed *vs* fasted animals, and at different times of the day, to demonstrate that FAA and anxiety are responses independent of fasting or daily photocycle. Finally, looking for neural mechanisms shared by both FAA and its linked-anxiety, and considering that the ghrelin antagonist reduces FAA in goldfish^34^, we ask whether it would also reduce anxiety-like state.

## Methods

### Animals

The animal model used was *Carassius auratus*, commonly known as goldfish, acquired from ICA S.A. (Madrid). Gender undifferentiated juvenile fish were housed in 60-Liter tanks (density around 1.5 g/L) with filtered and aerated water in the animal facilities of the University. They were kept under control conditions of temperature (21 °C) and 12L:12D photoperiod (12 h of light and 12 h of dark, lights on at 08:00, being this moment *Zeitgeber* Time 0, ZT 0). Granulated food (1.5% of body weight, bw; Sera Pond Biogranulat, Heisenberg, Germany) was provided daily through automatic feeders (Eheim, digital feeders #3581000) at 10:00 (ZT 2), unless otherwise specified in some experiments. Animals were acclimated to these conditions at least for two weeks prior to each experiment.

### Appropriate ethics declarations

The research was carried out in accordance with current European (EU63/2010) and the Spanish (RD53/2013) regulations and was approved by the Animal Experimentation Committee of the Complutense University of Madrid (PROEX 317.7/23) and by Comunidad de Madrid. All procedures as well as the methods description in this article follow the ARRIVE Guidelines 2.0^37^.

### Drugs and their administration

The ghrelin used in this study was synthesized by Bachem (Bubendorf, Switzerland) upon request, based on the goldfish ghrelin sequence: GTSFLSPAQKPQGRRPPRM, a 19-amino acid peptide acylated with an octanoyl group at the serine-3 position (GHR-S3). The ghrelin antagonists JMV2959 (ref: 345888, Merck Life Science, Madrid, Spain) and D-Lys^3^-GHRP-6 (D-lys; ref: G4535, Merck Life Science, Madrid, Spain) were used to block ghrelin receptor activity. Stock solutions of all compounds (1 mM) were prepared in ultrapure water and diluted to their final working concentrations—1 μM for ghrelin and 10 μM for both JMV2959 and D-lys—using teleost saline (600 mg NaCl and 15.8 mg NaHCO₃ in 100 ml of distilled water, pH 7.2). Aliquots were prepared and stored at − 80 °C until use.

Intraperitoneal (IP) injections were performed at a volume of 10 μl/g body weight (bw), corresponding to final doses of 10 pmol/g bw for ghrelin and 100 pmol/g bw for the antagonists, using the working solutions previously described (1 μM for ghrelin and 10 μM for both JMV2959 and D-lys). The doses of ghrelin and D-lys were selected based on previous teleost studies^34^, while the dose of the antagonist JMV2959 was based in rodent studies^38^. To minimize handling stress, goldfish were anesthetized by immersion in tricaine methanesulfonate (MS-222, 0.14 g/l) buffered with bicarbonate. Once anesthetized, injections were administered into the ventral midline, just posterior to the pelvic fins, using 1 ml syringes and 0.3 mm diameter needles. Following administration, fish were monitored to ensure full recovery before behavioral testing. Control groups in each experiment underwent the same handling and injection procedures but received an equivalent volume of teleost saline instead of the pharmacological compounds.

### Experimental designs

In order to explore whether the food-seeking behavior associated with FAA was an anxiety state, we conducted different experimental approaches (Supplementary Fig. S1) to answer the following four questions:

#### Is the FAA a behavioral state of anxiety?

In the initial experiment, fish weighing 4.2 ± 1.2 g bw (mean ± S.E.M. or standard error of the mean, N = 40) were acclimated to the standard 12L:12D photoperiod (lights on at 8:00 h) but fed at 13:00 h (ZT 5) to separate the increase in locomotor activity that occurs near the onset of light (ZT 0) from the food anticipatory activity. Anxiety–like status of the animals was analyzed sequentially by the open field and black and white background preference tests (as below described) during their FAA (at ZT 3.5–5 h) or after the FAA (at ZT 7–8.5). As indicated in Supplementary Fig. S1, this experimental protocol implies a fasting period of around 22.5–24-h for goldfish analyzed during their FAA (at ZT 3.5–5; group named FAA-24F-ZT 5; n = 20; Supplementary Fig. S1a), and 2–3.5-h postprandial for animals analyzed after the FAA (at ZT 7–8.5; group named PFAA-2FED-ZT 7; n = 20; Supplementary Fig. S1a).

#### Is the anxiety-like behavior found during FAA due to fasting?

Two approaches were conducted to ensure that the anxious state during FAA was due to anticipating feeding time and not a result of the possible acute anxiogenic or anxiolytic effect of fasting and feeding, respectively. All fish (6.9 ± 2.3 g bw, mean ± S.E.M. or standard error of the mean) were maintained at the above-described standard acclimation conditions (i.e. 12L:12D, light on at 8:00 h, and regularly fed at 10:00 h, ZT 2). In the first approach, behavioral tests were conducted sequentially on fasted fish during their expected FAA period (22.5–24-h of fasting at ZT 2, named FAA-24F-ZT 2, n = 14, Supplementary Fig. S1b) or 6 h later 29.5–31-h of fasting (PFAA-30F-ZT 8; n = 14; Supplementary Fig. S1b). In a complementary experiment to discard an acute anxiolytic effect of feeding, anxiety behavior was measured in a group (n = 16) of fasted fish not coinciding with their FAA (after 29.5–31-h of fasting, named PFAA-30F-ZT 8; Supplementary Fig. S1c), and in a group of fed fish 2–3.5-h after feeding. On the day of the experiment this later group received the food at ZT 8 (n = 15, named PFAA-2FED-ZT 10, Supplementary Fig. S1c).

#### Does the anxiety-like state reflect a putative daily rhythm driven by the daily light–dark cycle?

Another experiment was conducted to discard the possibility that the differences found in the previous experiments might be due to a putative daily rhythm in anxious behavior (since the trials were performed at different times of the day). Animals (8.8 ± 2.4 g bw; mean ± S.E.M. or standard error of the mean, N = 27) were maintained under constant light (i.e. in the absence of a daily light–dark cycle) and were fed at 10:00 h, as showed in Supplementary Fig. S1d. Behavioral tests were conducted sequentially at 22.5–24 h of fasting (coinciding with FAA, LLFAA-24F, n = 13), and 2–3.5 h post-feeding (at 12:00 h, LLPFAA-2FED, n = 14).

#### Do ghrelin antagonists have an impact on anxiety-like behavior?

To investigate whether endogenous ghrelin was involved in the anxiety-like response during the FAA, two antagonists of the hormone were employed. Goldfish (N = 27, 7.8 ± 2.1 g bw, mean ± S.E.M. or standard error of the mean) acclimated at 12:12D and fed at ZT 2 (lights on at 8:00 h) were divided into three experimental groups (n = 9/group): control, treated with the ghrelin antagonists JMV2979, and treated with the ghrelin antagonist D-lys. The day of the experiment, at ZT 1 (during their FAA) vehicle (teleost saline) alone or containing the ghrelin antagonists (JMV2959 and D-lys; 100 pmol/g bw) were administered intraperitoneally (IP) (Supplementary Fig. S1e). After 1 h post-injection (ZT 2), the open field and the black and white background preference tests were performed sequentially.

#### Does ghrelin induce anxiety-like behavior after FAA, and can its antagonist reverse it?

To study if ghrelin itself had anxiety-like properties, 36 goldfish (9.8 ± 2.3 g bw) were divided into four experimental groups and injected 2–3 h postprandially (when endogenous ghrelin levels are low^39^): Control (C, n = 10), injected twice with saline (teleost saline) at a 10 min interval; D-lys (D, n = 8), injected first with the antagonist and, after 10 min, with saline; ghrelin (G, n = 8), injected first with saline and then with ghrelin after 10 min; and the double-treated group (D-G, n = 10), injected first with D-lys and then with ghrelin after 10 min (Supplementary Fig. S1f.). One hour after the second intraperitoneal injection, the open field and black-white tests were performed.

To verify that the batch of commercially synthesized ghrelin (a 19-amino acid peptide acylated with an octanoyl group at the serine-3 position, as detailed below) had the expected orexigenic function at the dose employed, a feed intake test was conducted. Goldfish of 4.5 ± 0.2 g bw were divided into two groups (n = 10/group): Control (C, injected with teleost saline) and ghrelin (G, treated IP with ghrelin, 10 pmol/g bw). The feed intake was measured in satiated animals (2 h postprandially) for 2 h, as previously described^40^.

### Locomotor activity

Locomotor activity was recorded during acclimation in all experiments previously described to confirm the presence of FAA in all groups prior to conducting the behavioral tests. Six photocells were fixed to the tanks: two below the automatic feeders, and the remaining four at a height of 2–18 cm above the bottom of each aquarium wall. To minimize external interference, the aquarium walls were covered with opaque paper. Each time a fish moved in front of a photocell (Omron, E3S-AD12), the beam of light emitted by the sensor was interrupted, resulting in the generation of a pulse. The sensors were connected to an actimeter, which transmitted the number of light beam interruptions every 10 min to the data acquisition software Adq16 (a non-commercial software generated by Micronec for our internal use, Madrid, Spain). The software El Temps (v.313; www.el-temps.com) was used to generate profiles of average daily activity rhythms, actograms, and periodograms. Chi-square periodograms were employed to identify significant periods (*p* < 0.05) in activity rhythms, as outlined in previous works^35^.

### Behavioral tests

Anxiety-like behavioral tests were performed following the protocols previously validated in our laboratory^36^.

#### Open field test

A home-made 50-cm diameter circular acrylic aquarium was utilized for this test. The aquarium had a white bottom and opaque black walls. An object (a yellow and blue toy of 7 cm in height and 4 cm in diameter) was placed in the center for the fish to explore. Three zones were established: the wall zone (occupying 25% of the total area and near the walls), the open field zone (occupying 75% of the total area and situated in the inner part of the aquarium), and the center zone (occupying 25% of the total area and containing the novel object). The water depth was 10 cm. The trials were performed in a controlled environment with a temperature of 21 °C and illumination between 200 and 220 lx.

The protocol, as previously described^36^, involves capturing the fish with a net and transferring it to the testing room in a 5L tank, then allowing it to acclimate for 5 min. Subsequently, the fish is introduced into the tank close to the wall in the same position for each experiment. The movement of the fish is recorded for 10 min using a video camera positioned approximately half a meter above the tank. Following the recording, the videos are analyzed using EthoVision XT17 software by Noldus (Wageningen, The Netherlands). The parameters evaluated include average velocity in the open field, time spent near the wall and in the open field, latency to enter the open field and central zones, and the number of entries into the open and central zones.

#### Black and white background preference test

A rectangular tank with opaque walls and bottom (Maze Engineers) was employed to conduct a test of scototaxis following previously described protocols^36^. The tank is divided into two halves, one of which is white and the other black. The dimensions of the tank were 47 cm in length, 10 cm in width, and 14.5 cm in height, with a water depth of 10 cm. The animals were moved from the room where the open field test took place to the experimental room for this test in 5L tanks individually and allowed them to acclimate for 5 min before being placed on the black side of the tank. Behavioral data were acquired through automated tracking of the videos using Ethovision XT 17 software (Noldus). The parameters analyzed included latency to enter the white zone, the number of crossings between the black and white zones, and the time spent in the white zone. The temperature (21 °C) and illumination levels (600–650 lx) were maintained at a consistent level within the room.

### Statistical analysis

SigmaPot 12.0 was used for the statistical analysis and representation of the behavioral data obtained during the experiments. The Student T-test (two tailed) test was used to detect the differences between two groups. For FAA analysis, the mean of locomotor activity in the 2 h prior to feeding time was compared with the mean of the activity each 2 h throughout the rest of the day in each experimental group for all days represented. All behavioral variables measured were also compared by a T-test. In the experiment combining ghrelin and its antagonist, a two-way analysis of variance (ANOVA) was conducted to examine the effects of ghrelin and D-lys, as well as their interaction. When a significant interaction was found, a one-way ANOVA followed by Fisher’s LSD post hoc test was performed for pairwise comparisons. The normality and homoscedasticity conditions of the data were verified by using the Shapiro–Wilk and Levene tests; in case of non-compliance, the data were transformed to the square root or to the logarithm with base 10.

## Results

### Fish showed FAA in all experimental acclimation conditions

**Fig. 1:**
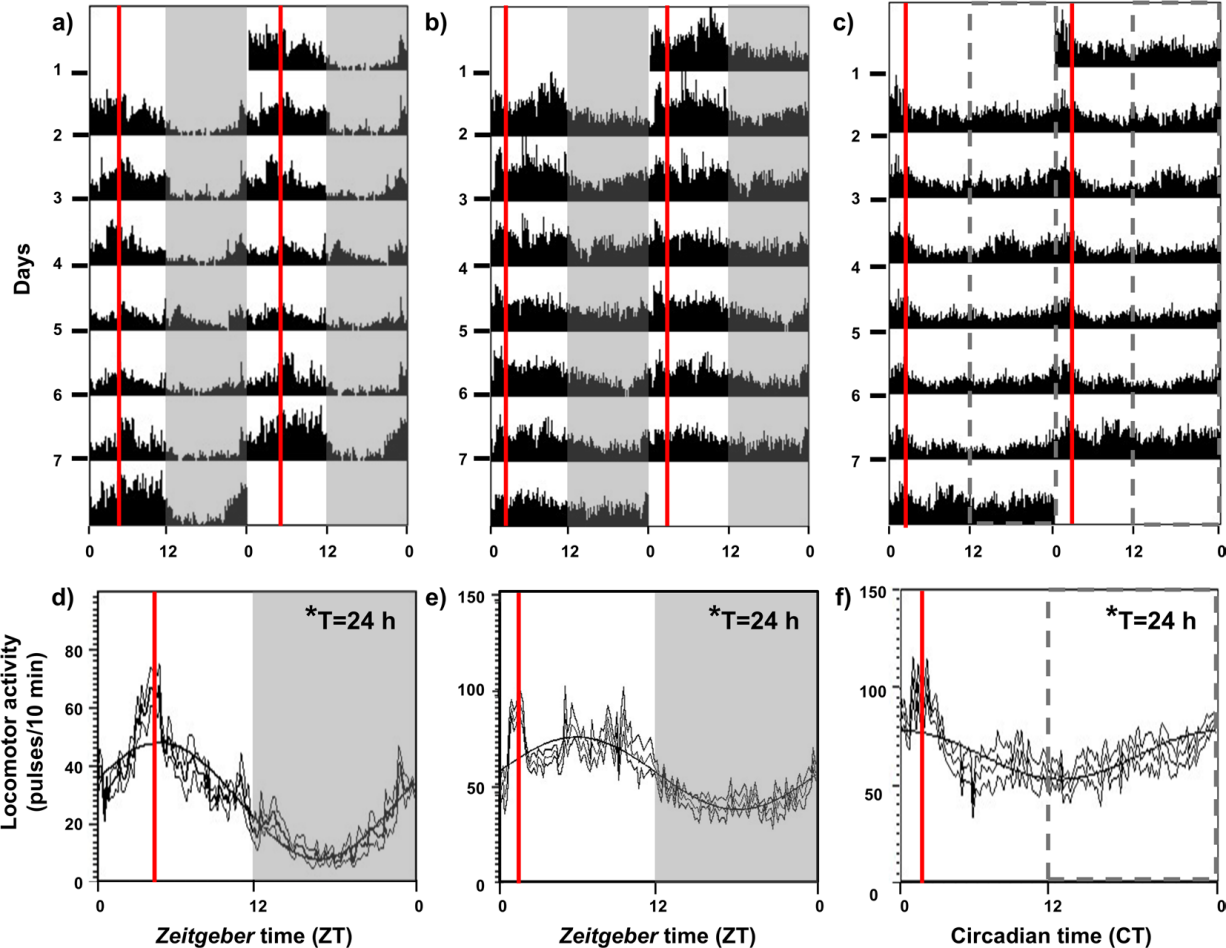
Representative actograms of the locomotor activity of goldfish over a 7-day period before any experiment. The fish were fed daily at either 13:00 h (**a**) or 10:00 h (**b**) under a photoperiod of 12L:12D or were fed daily at 10:00 h under constant light (**c**). The actograms are plotted on a 48-h time scale (double plotted). Average waveform of locomotor activity for each group (**d**–**f**) was calculated from 7 consecutive days (mean ± SEM). The white areas of the graphs correspond to the light phase (08:00–20:00, ZT 0-ZT 12), while the grey areas correspond to the dark phase (20:00–08:00, ZT 12-ZT 0) of the daily photoperiod. The feeding time (10:00 or 13:00 h, ZT 2, ZT 5 or CT 2) is indicated by the vertical red line.

Representative locomotor activity actograms and waveforms of fish fed at either 13:00 or 10:00 h under a photoperiod of 12L:12D and fish fed at 10:00 h under constant light are presented in Fig. 1. Activity recordings were performed to ensure that all animals were synchronized to the light–dark cycle and feeding time and exhibited FAA at the expected hours in all experiments. The actograms and waveforms demonstrated that the fish exhibited a significant circadian rhythm of 24 h (Fig. 1a–f), with higher activity during the light phase compared to the dark phase (Fig. 1a,b,d,e). However, the group under constant light showed a lesser difference between these phases (Fig. 1c,f). All groups of animals exhibited synchronized feeding behavior, as evidenced by the rise in locomotor activity approximately two hours prior to feeding. The locomotor activity during the 2 h previous to expected feeding time in the animals under 12L:12D and schedule fed at 13:00 in the 7 days before the experiment was 669.4 ± 53.8 pulses, while the mean during the rest of the day was 350 ± 22.9 pulses/each 2 h (T = 5.460, *p* = 0.0001). Goldfish under 12L:12D and feeding schedule at 10:00, showed 907 ± 32 pulses (from 8:00 to 10:00), while the mean in the rest of the day was 680 ± 24 pulses each 2 h in (T = 5.598, *p* = 0.0001). Finally in the third experimental acclimation conditions (under LL and schedule fed at 10:00) activity from 8:00 to 10:00 was also higher than the mean of the resto of the day (1095 ± 75 vs 719 ± 54 pulses each 2 h; T = 4.490, *p* = 0.0007). This pattern aligns with the expected timing of FAA in each group, according to their experimental conditions.

### Fish in the FAA period showed anxiety-like behavior

**Fig. 2:**
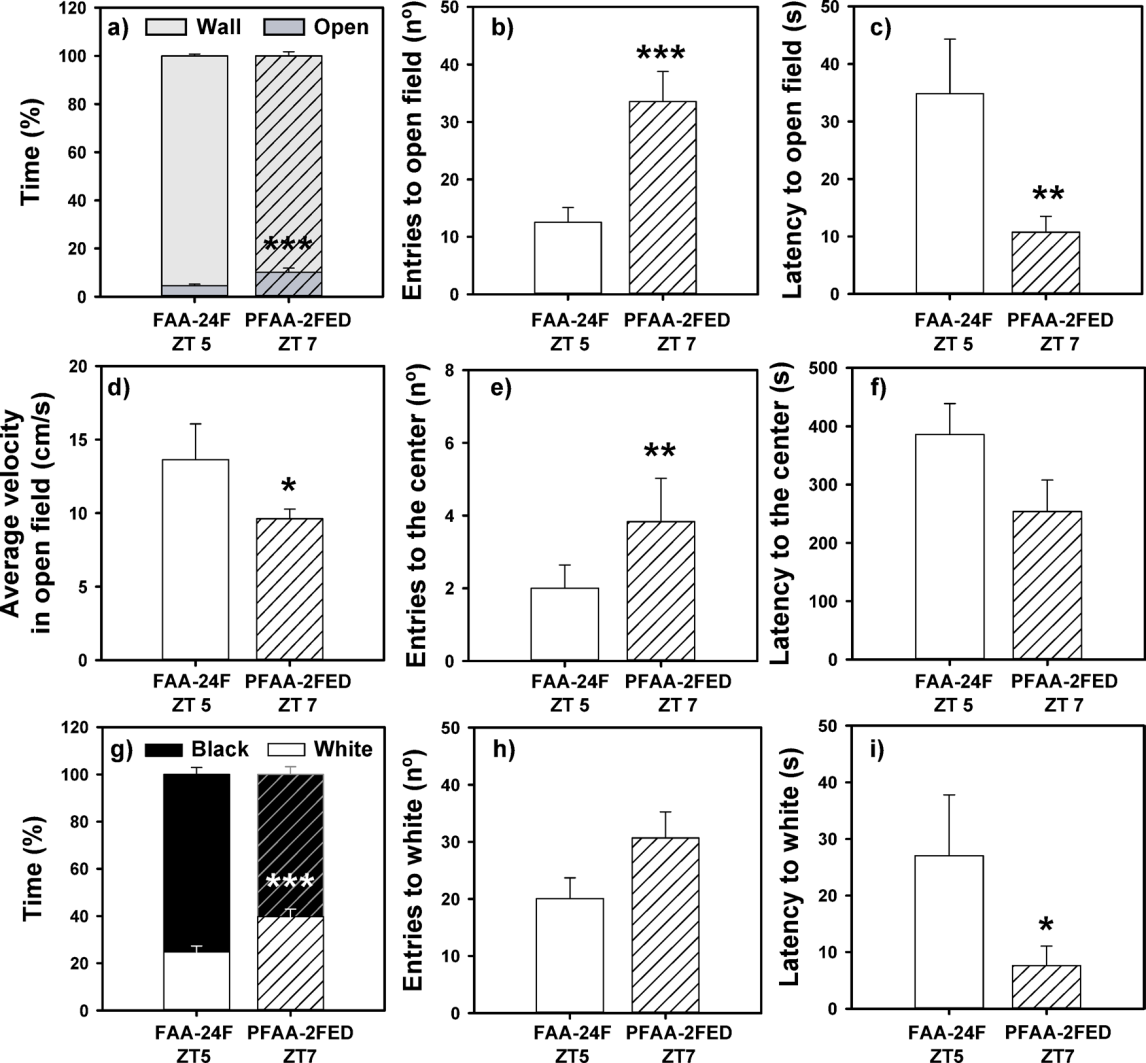
Open field (**a**–**f**) and black and white preference (**g**–**i**) tests in goldfish in the FAA period (24-h fasting) or post-FAA period (2-h post-prandial). Mean values + standard error (n = 20) are represented. Student T-test *p < 0.05, ***p < 0.001. FAA, food anticipatory activity; PFAA, post-food anticipatory activity; 24F, 24-h of fasting; ZT, *Zeitgeber* Time.

The animals that were at the moment of expected FAA exhibited higher thigmotaxis and scototaxis compared with the fed fish that were in the post-FAA period, as can be seen in the heatmaps (Supplementary Fig. S2). The FAA group (compared with animals who were past their FAA phase and had eaten) spent less time in the open field (Fig. 2a), entered fewer times there (Fig. 2b), took more time to enter the first time (latency, Fig. 2c), and showed higher velocity in this area (Fig. 2d; statistical data in Supplementary Table S1). These animals also tended (not significantly) to enter less in the center (Fig. 2e) and to have higher latency to enter this zone (Fig. 2f). In accordance with these results, FAA-24F animals spent less time in the white area in the black and white background preference test (Fig. 2g), accompanied by a significantly longer latency time (Fig. 2i, statistical data in Supplementary Table S1), when compared with fish tested after their FAA phase. The number of entries in white tended, congruently, to be lower in fish during FAA (Fig. 2h), but it was not significantly different.

### FAA-anxious state was not dependent on fasting hours

**Fig. 3:**
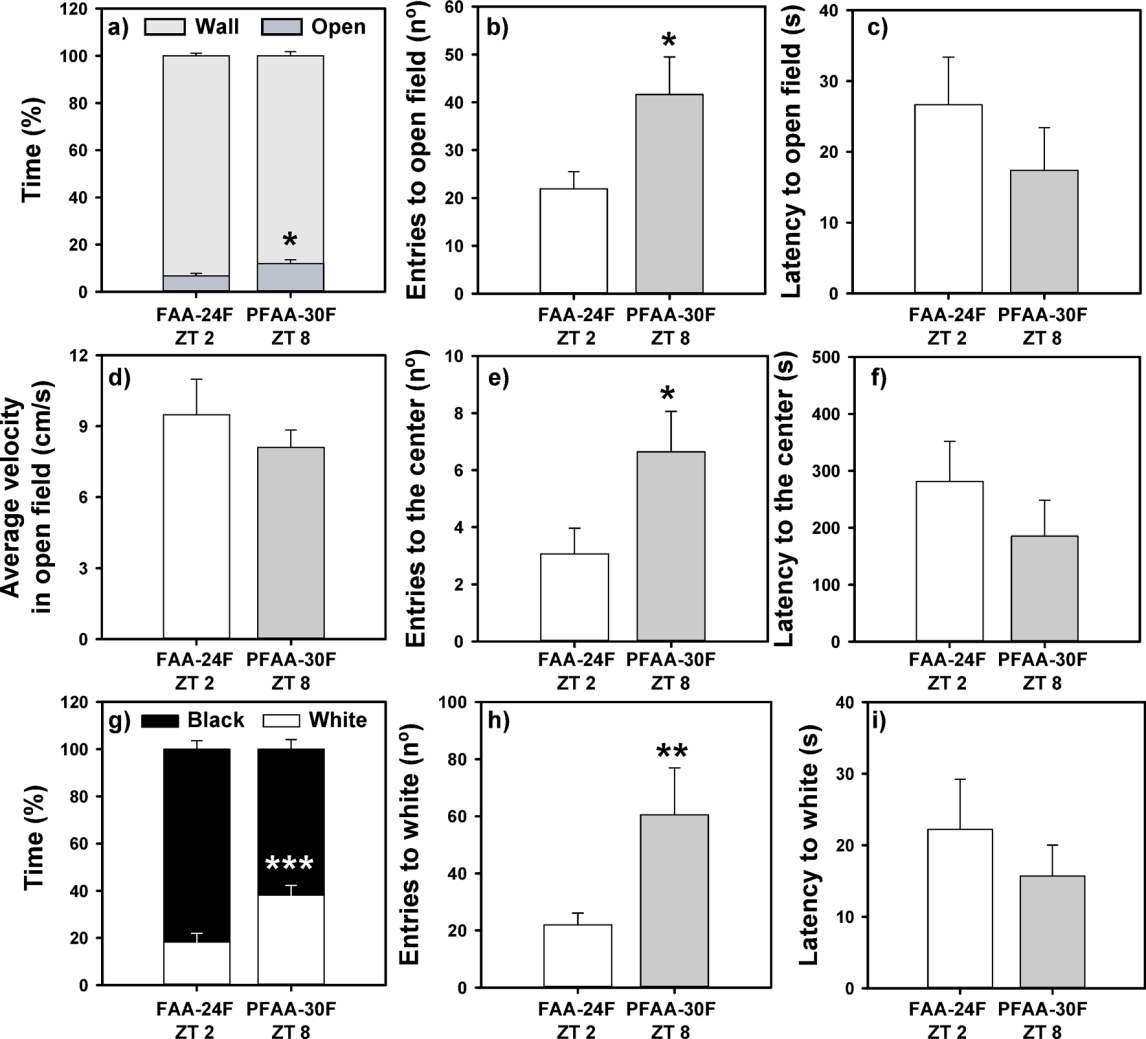
Open field (**a**–**f**) and black and white preference (**g**–**i**) tests in goldfish in the FAA and 24-h fasting or in the post-FAA period after 30-h fasting. Mean values + standard error (n = 14) are represented. Student T-test *p < 0.05, **p < 0.01, ***p < 0.001. FAA, food anticipatory activity; PFAA, post-food anticipatory activity; 24F, 24-h of fasting; 30F 30-h of fasting. ZT: *Zeitgeber* Time.

The animals that had been fasting for 30 h but were in the post-FAA showed lower anxiety-like behavior than during FAA phase (see heat maps in Supplementary Fig. S3), as indicated in Fig. 3, and confirmed by statistics (Supplementary Table S2). Fish that had fasted for 30 h spent a greater proportion of time in the open area and made more entries to the open field and center than those that had fasted for 24 h but were tested in the FAA phase (Fig. 3a,b,e; Supplementary Table S2). The latency to enter into the open field (Fig. 3c) and the center zone (Fig. 3f) tended to be higher in the 24F *vs* 30F goldfish although there were not statistically significant differences. A comparable outcome was observed in the black and white preference test. In this instance, FAA animals that had fasted for 24 h demonstrated a heightened degree of scototactic behavior, as evidenced by the greater proportion of time spent in the black zone and the fewer entries into the white zone (Fig. 3g,h; Supplementary Table S2). The latency to enter into the white aversive zone tends to be higher in 24F versus 30F animals (Fig. 3i) but there were no statistically significant differences.

### Eating is not anxiolytic per se

**Fig. 4:**
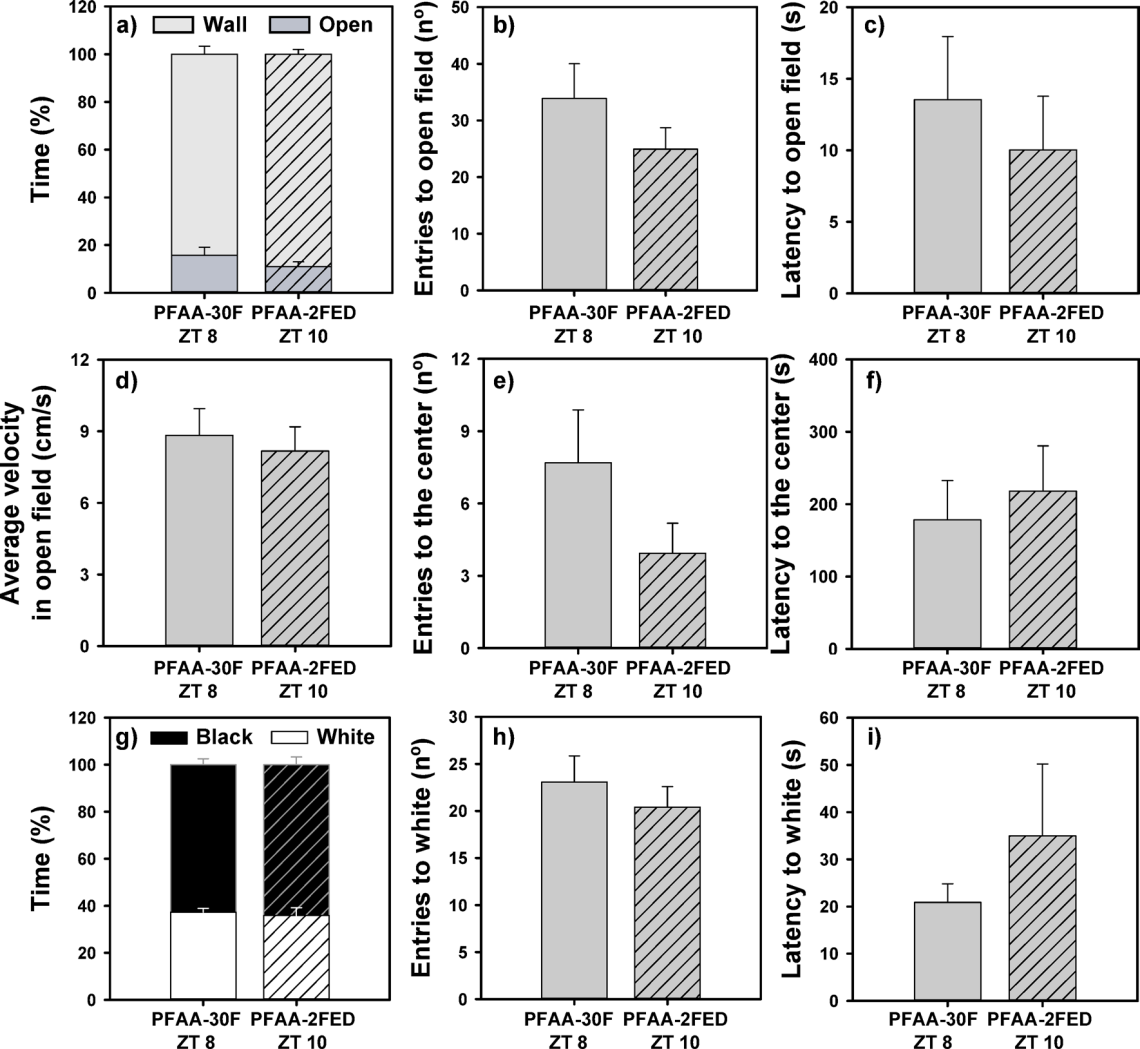
Open field (**a**–**f**) and black and white preference (**g**–**i**) test in goldfish after the post-FAA period after 30-h fasting or 2-h post-prandial. Mean values + standard error (n = 15–16) are represented. PFAA, post-food anticipatory activity; 30F, 30-h fasting; ZT: *Zeitgeber* Time.

Although all animals presented aversion for open field and white zones as shown in heatmaps (Supplementary Fig. S4), there were no significant differences between the two groups in none of the parameters analyzed in the open field test or the black and white test (Fig. 4; Supplementary Table S3). Thus, fish displayed similar anxiety-like behavior, although one group was in 30-h fasting while the other received a meal 2-h before.

### Anxiety-like behavior caused by FAA is not dependent on a light–dark cycle

**Fig. 5:**
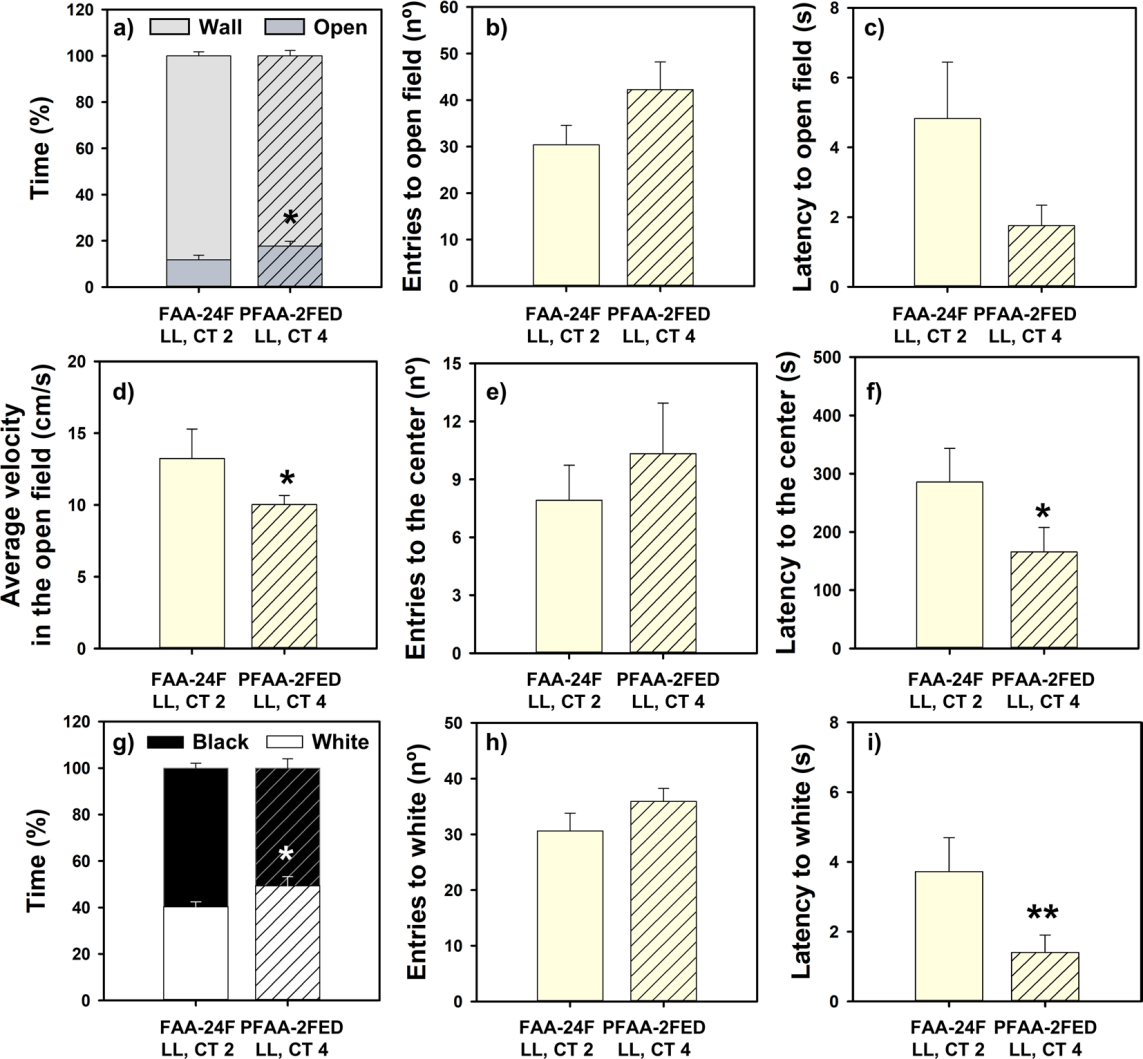
Open field test (**a**–**f**) and black and white preference (**g**–**i**) in goldfish in the FAA period (24-h fasting) or post-FAA period (2-h post-prandial) in animals under constant light. Mean values + standard error (n = 13–14) are represented. Student T-test *p < 0.05, **p < 0.01. FAA, food anticipatory activity; LL, constant light; PFAA, post-food anticipatory activity; 24F, 24-h of fasting; CT: Circadian Time.

In the absence of a light–dark cycle, fish during the FAA period spent less time in the aversive zones (open field or white) as shown in the heatmaps (Supplementary Fig. S5), suggesting that they were more anxious than after FAA period. This was also evidenced by an increment of the time spent near the wall area (Fig. 5a), the swimming velocity in the open zone (Fig. 5d). The time taken to enter the open field compared to the fed group tended to increase during the FAA moment (Fig. 5c; Supplementary Table S4). The numbers of entries in aversive zones, open field and center, tended to be lower in the fasted *vs* fed group (Fig. 5b,e), but with no statistically significant differences, as occurs for latency to the center that only tend to increase in fasted *vs* fed animals (Fig. 5f).

Consistently, in the black-white test, the FAA group demonstrated a reduced percentage of time spent in the white area (Fig. 5g) and an increase in the latency time to enter the white area (Fig. 5i), without any significant changes in the number of entries in the white zone (Fig. 5h; Supplementary Table S4).

### The ghrelin antagonists reduced the anxiety-like behavior

**Fig. 6:**
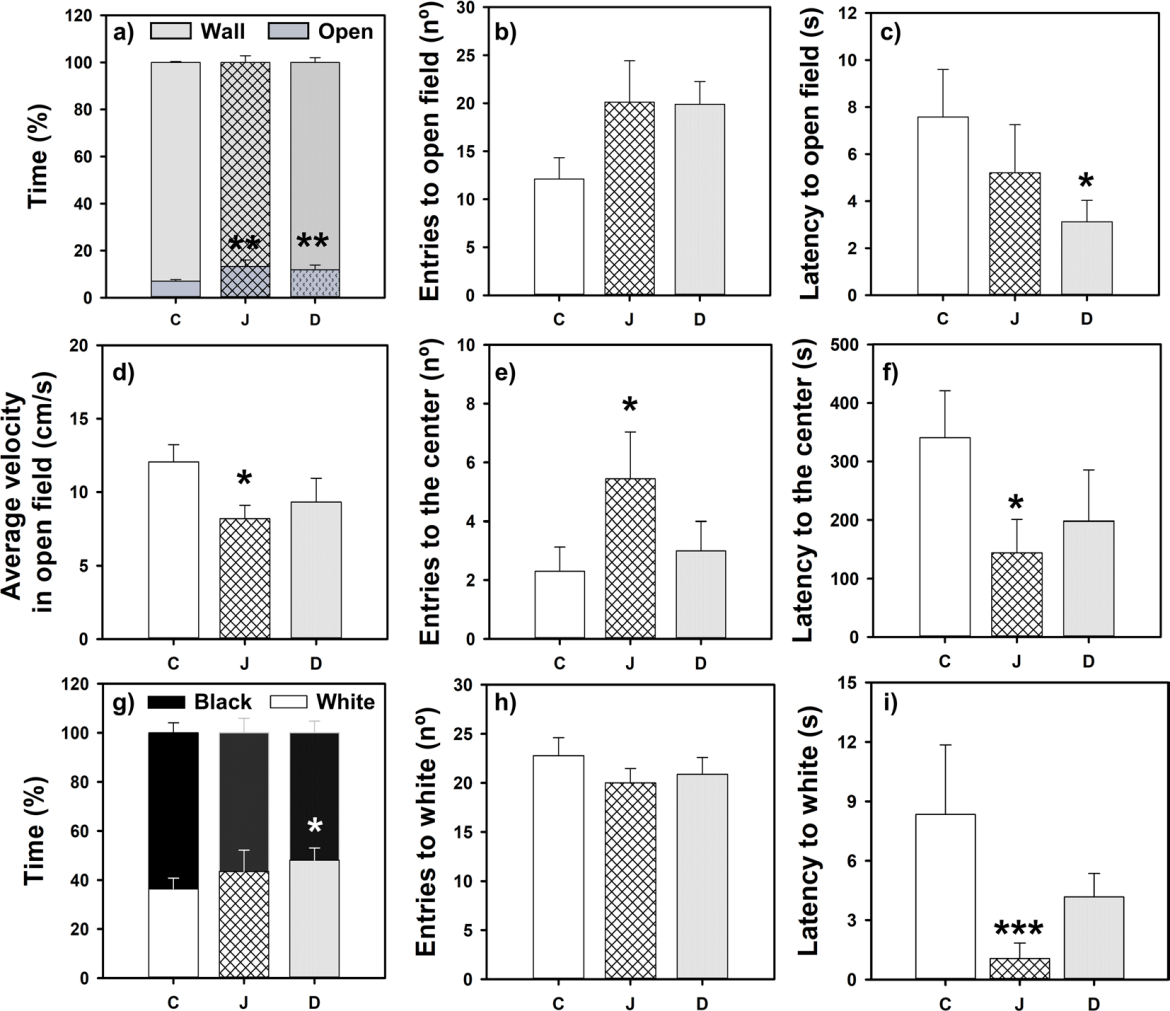
Effect of IP treatment with ghrelin antagonists in the open field (**a**–**f**) and black and white background preference (**g**–**i**) test in goldfish during the FAA. Mean values + standard error (n = 9) are represented. Student T-test *p < 0.05; **p < 0.01. Control group (C), and ghrelin antagonists: JMV2959 (J) and D-lys (D).

The acute intraperitoneal (IP) administration of the ghrelin antagonists JMV2959 and D-lys reduced anxiety-like behavior in goldfish during the FAA period. Both treatments decreased thigmotaxis in the open field test, as the animals spent less time near the walls (Fig. 6a; Supplementary Fig. S6; Supplementary Table S5–S6). Fish treated with JMV2959 ventured more frequently into the center and exhibited lower velocity in the open zone compared to vehicle-treated controls (Fig. 6d,e). D-lys significantly decreased the latency to enter the open zone, while JMV2959 reduced the latency to enter the center (Fig. 6c,f). Although not statistically significant, both JMV2959 and D-lys increased the number of entries into the open zone (Fig. 6b). In the black and white preference test, D-lys significantly increased the percentage of time spent in the white compartment (Fig. 6g). However, there were no significant differences in the number of entries into this area between groups. The treatment with MV2959 reduced the latency to enter the white compartment but had no significant effect on the percentage of time spent in white (Figs. 6g–i).

### Ghrelin increased anxiety-like behavior 

First, obtained results validate the effectiveness of the peptide employed (19 aa, GHR-S3) at the dose of 10 pmol/g bw as orexigenic, as ghrelin treated fishes showed a significant (*p* < 0.05) increase in feed intake compared to the control group (13.28 ± 1.55 mg/g bw *vs* 7.31 ± 1.58 mg/g bw; Supplementary Fig. S7).

**Fig. 7:**
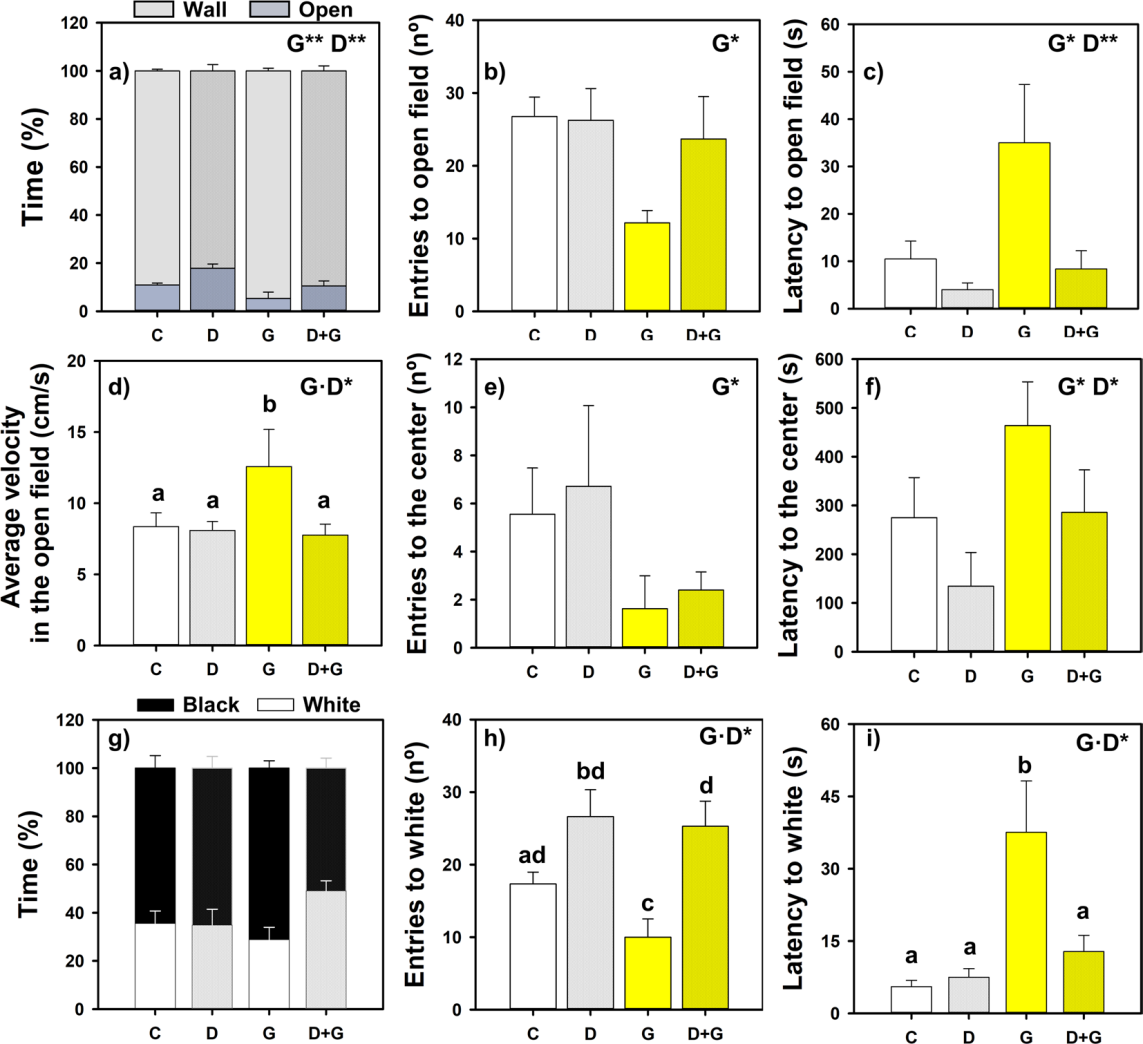
Effects of IP treatment with ghrelin and its antagonist (D-Lys) in the open field (**a**–**f**) and black and white background preference (**g**–**i**) test in goldfish. Means + standard errors (n = 8–10) are shown. Two-way ANOVA was performed, G: significant effect of ghrelin (*p* < 0.05), D: significant effect of D-lys (*p* < 0.05), G·D: significant interaction (*p* < 0.05), *p < 0.05. In case of interaction between factors a post-hoc was performed, different letters indicate significant differences (*p* < 0.05) by Fisher LSD test. G is the ghrelin factor, and D is the D-lys factor. Control group (C), D-lys group (D), Ghrelin group (G) and D-lys with Ghrelin group (D + G).

Regarding ghrelin effects on anxiety-like responses, results obtained in the open field test in animals treated with ghrelin alone and combined with and its antagonist (D-lys) are shown in Fig. 7, supporting an anxiogenic role of ghrelin that is reversed by its antagonist. Treatment with ghrelin significantly decreased the percentage of time spent in the open field (Fig. 7a), while increasing the latency to enter both aversive zones, the open field and the center (Fig. 7c,f; Supplementary Fig. S8; Supplementary Table S7). Additionally, ghrelin reduced the number of entries into both areas (Fig. 7b,e). Moreover, D-lys administration significantly increased the percentage of time spent in the open field (Fig. 7a) and also decreased the latency to enter it (Fig. 7c). Ghrelin-treated group animals also exhibited higher velocity in the open zone compared to all other groups (Fig. 7d).

Results of the black and white preference test showed that ghrelin decreased the number of entries into the white compartment, while it significantly increased the latency to enter this area (Fig. 7h,i)—both effects indicative of heightened anxiety-like behavior (Supplementary Fig. S8; Supplementary Table S6). No significant differences were observed in the percentage of time spent in the white zone, although ghrelin tends to decrease it (Fig. 7g).

## Discussion

The present study demonstrates that the food anticipatory activity is an anxiety-like state in goldfish, which is not a reflection of a putative basal daily rhythm in anxiety. This anxiety linked to FAA is not just mediated by either fasting or related to a putative anxiolytic effect of meal, and probably reflects a physiological arousal due to the expectation of the upcoming food reward.

Actograms indicate that goldfish showed daily locomotor activity rhythms with higher activity during the photophase when maintained at 12L:12D photoperiod. This aligns with the diurnal nature of the species under such conditions previously reported^32,41,42^. Additionally, the FAA is present regardless of whether feeding time was at 10:00 or 13:00, suggesting that mealtime controls the anticipation rather than the moment of day, as formerly postulated^13,33^. Even in the absence of a daily photocycle (i.e. constant light), the FAA is maintained when animals were fed with a time restricted feeding schedule, as expected in this species^43,44^. This confirms that FAA in goldfish is regulated by a FEO, as previously demonstrated in this species ^32,41^ and expected for a food-entrained rhythm^9,16^, supporting that this species is an exceptional model to study food-anticipatory behavior^3^. Moreover, locomotor activity recordings allowed us to know the optimal timing for performing behavioral tests of anxiety responses, by controlling the time window when animals exhibit FAA in each experimental design.

The tests used to measure mainly thigmotaxis and exploration (open field with novel object) and scototaxis (white-black preference), as indicators of anxiety-like behaviors, have been previously optimized for goldfish^36^, adapting tests commonly employed in zebrafish^45–47^. The open field test evaluates the preference for wall versus open spaces^47^. The primary indicator for quantifying anxiety is the "total open area avoidance" (encompassing both open space and center in this study). Additionally, this experiment measures the animal’s exploration capacity by introducing a novel object in the tank’s center, assessing neophobia or unfamiliarity aversion. If the animal exhibits low anxiety levels, it will attempt to enter the open area and to explore the object^36,46^. The black-white background preference test enables evaluation of an animal’s scototaxis or preference for the black area, known to be perceived as a safer zone for multiple species of fish and mammals^48^. In the present research goldfish from all studied experimental groups spent more time in the near-wall and black areas, supporting the strong thigmotactic and scototactic behavior in this species^48,49^. It also evidences the useful employment of these non-invasive techniques to explore welfare in fish together alongside range of other methods^50^, although individual personalities in anxiety responses have been described in fish^36,51^.

Present data clearly show that goldfish during their FAA period (FAA24F ZT 5 group) displayed higher levels of anxiety compared with fish at 2-h postprandial (PFAA2FED ZT 7 group). Animals that were tested during the time preceding an expected meal spent less time in the open and white aversive zones and exhibited greater neophobia (higher latency to enter these zones). Moreover, the swimming velocity in the aversive open region was also higher, which indicates a higher level of discomfort and a desire to return to the safe region near the wall ^46^. However, although FAA is associated with anxiety-like behavior and post-feeding animals (after their FAA) show a more relaxed phenotype, it could be proposed that animals are anxious because they are hungry (due to the restricted feeding schedule, approximately 24 h of fasting), or maybe that a recent meal in the PFAA2FED could have an anxiolytic effect. The results of our second experiment aimed to discriminate expectation of food arrival from fasting and allowed us to discard both proposals. It appears that fasting alone cannot explain the observed anxious state. Fish fasted for 24 h (FAA24F ZT2 group) display higher thigmotaxis and scototaxis responses in the open field and black and white tests than goldfish after a 30-h fasting (PFAA30F ZT8 group), again pointing to an increased anxiety linked to the anticipation of a meal and not to fasting. On the other hand, when fish were tested after their FAA, no significant differences were found between the fish fasted for 30 h and those fed 2 h later (PFAA30F ZT8 *vs* PFAA2FED ZT10), neither in the open field nor in the black and white test. This evidences that food intake alone is not enough to produce an anxiolytic postprandial response, probably because reward it is not the same when food arrives at an unexpected time. It has recently been proposed that goldfish with a randomly-timed-feeding protocol are more anxious than fish with scheduled meals^36^, and in fact this fish eat more^52^, probably because the hedonic system is not functioning correctly. In this sense, FAA in fish is probably more related to hedonic control rather than homeostatic control of feeding, as suggested in mammals^10,16^, what would explain why rats fed ad libitum with regular chow display FAA if offered a palatable meal at restricted schedule times^19,25^.

Up to here, the data discussed implies that anxiety-like behavior coincides in time with FAA, what we link with the expectation of food reward at a specific time temporal window^10,16^, however it could be also caused by rhythms in anxiety-like behavior linked to photocycle. Recently a daily rhythmicity in thigmotactic response using the open field test has been described in zebrafish, with reduced anxiety states during daytime^31^. Authors correlate such rhythms with endogenous oscillators entrained by the photocycle, but they do not study FAA and these animals were fed twice a day. Thus, a new question emerges—are the anxiety-like behavioral responses observed in the present work linked to photocycle? To address this matter, goldfish were exposed to a constant dim light (LL), with a fixed feeding schedule. As previously mentioned, these animals still display food anticipatory activity, as expected^33,43^. Differences in anxiety-like behavior between the FAA and post-FAA groups also persisted, as FAA-animals entered less often the aversive zones (open and white). This suggests that the light–dark cycle is not necessary for FAA-related anxiety-like behavior, supporting again that both responses are mediated by a FEO and not by a LEO. It is noteworthy that, although statistically significant, differences in the variables studied between both groups in these experiments are less pronounced than in previous ones. This may be because LL conditions produce a relatively higher anxiety baseline in these animals^36^, as a result of chronodisruption, a stressful dysregulation of the circadian system described in fish, birds, and mammals^53–55^.

Overall, up to this point, the results indicate that FAA and anxiety in goldfish are strongly linked, and there may be common neurobiological mechanisms underlying both behaviors. Most studies that investigate central mechanisms involved in food reward in fish quantify food intake and do not allow to separate the involvement of the homeostatic and the hedonic feeding control^56–58^. Thus, present results are relevant because they suggest that the anxiety-like behavior (such as the one observed during food anticipation) in this model of schedule-restricted feeding could be used to deepen our understanding of the fish hedonic system, especially regarding food reward responses, whose knowledge is limited in fish to date^6^. The use of palatable diets to study their influence on anxiety, as has been shown for FAA^18,19,25^, could also be useful to support the link with the hedonic system. To further explore this hypothesis and identify hedonic signals involved in the observed anxiety responses, we performed the next experiment, in which two ghrelin antagonists were administered to goldfish during their FAA. Ghrelin has been associated with the onset of FAA in both mammals^59,60^ and fish^34^. Moreover, a pre-prandial peak in ghrelin has been observed in goldfish^39^. Thus, if anxiety was induced by an FAA mechanism, it was expected that the antagonists would have an anxiolytic effect. The results confirm this, since fish injected with both ghrelin antagonists displayed reduced anxiety levels compared to the control group in both the open field and black-and-white tests, again pointing to shared mechanisms for FAA and its associated anxiety behavior in goldfish. Based on all these findings, it can be also proposed that ghrelin induces an anxiety-like state in the teleost fish, as in mammals^61,62^. Our last experiment confirms such a role of ghrelin. Ghrelin injected after a meal (i.e. during relaxed state) induces anxiety, which was at least partially reversed by the ghrelin antagonist.

In this sense, it has been shown that, at least in rodents, ghrelin intervenes centrally in more complex aspects related to ingestion, such as learning, motivation and anxiety, acting on the hippocampus, amygdala and dorsal raphe nucleus^63–65^, or on the accompanying hedonistic aspects, activating mesolimbic reward centers^63,66–68^. Thus, we suggest that ghrelin could be involved also in the hedonic system in fish what explained its effect on FAA^34^ and on anxiety (present data). However, although we cannot discard that ghrelin effects on anxiety could be mediated by homeostatic hypothalamic ghrelin targets that in fish are also well known, such as orexin or NPY^69^, that could contribute to anxiety. Many arguments support the first hypothesis, in mammals, ghrelin has been associated with enhanced palatability and increased anticipatory activity in response to the consumption of palatable foods^25,70^. Moreover, some studies have indicated that the stimulatory effects on pleasurable reactions and the triggering of the corticolimbic reward-cognitive systems when evaluating food are similar between ghrelin administration and fasting^71^. All these data confirms that ghrelin is part of the hedonic system in mammals. Although its role in this system in fish is not fully understood, the previously mentioned results in goldfish show that ghrelin increases food intake^72^, exhibits a pre-prandial peak coinciding with FAA^39^, and that a ghrelin antagonist interferes with the onset of FAA^34^. This suggests that the anxiety associated with FAA and the anxiolytic effect of the ghrelin antagonist could be explained by ghrelin’s involvement in the hedonic system or through additional mediators of this system that also trigger FAA. In this context, dopamine is a strong candidate, as it is involved in reward and anxiety responses in both mammals^73,74^ and fish^75^, and is a primary mediator of reward responses in mammals^76,77^.

Furthermore, in mammals, ghrelin has been shown to act on the ventral tegmental area (VTA), inducing dopamine release in the nucleus accumbens, thereby promoting reward-related behaviors and modulating food motivation^78,79^. Although this mesolimbic pathway has not been anatomically described in teleost fish, dopamine-containing neurons located in the diencephalon project to the telencephalon and have been proposed as a functional homologue of the mammalian mesolimbic system^80,81^. Notably, in lungfish—sarcopterygians more closely related to tetrapods—a mesostriatal dopaminergic pathway originating in the VTA has been described, suggesting that this circuit may have emerged early in vertebrate evolution^82^. In this context, investigating the presence of ghrelin receptors in diencephalic dopaminergic neurons of teleosts, and the potential for ghrelin to trigger dopamine release, could provide valuable insights into the motivational circuitry underlying FAA.

## Conclusions

Overall, present data clearly demonstrates our hypothesis that FAA is an anxious state in goldfish, not directly mediated by fasting nor food intake, but by a food-entrained oscillator that links both behaviors and determines when these responses are triggered. In addition, we propose that these responses are mediated by the hedonic system in fish, involving the action of ghrelin. The possible involvement of ghrelin in activating dopamine-reward circuits in teleosts, should be explored in future studies to gain further insight into the neurobiological basis of food anticipatory activity.

## Supplementary Information

Below is the link to the electronic supplementary material.

Supplementary Material 1

## Data Availability

Data are free available in the Open Access Institutional Repository of the Complutense University of Madrid, DOCTA (https://hdl.handle.net/20.500.14352/109091).
