# Correction: Food reward entrainment increases mealtime anxiety in goldfish via a ghrelin-dependent mechanism

**DOI:** 10.1038/s41598-026-59849-1

**Published:** 2026-06-26

**Authors:** Lisbeth Herrera-Castillo, Nuria Saiz, Nuria de Pedro, Esther Isorna

**Affiliations:** Department of Genetics, Physiology and Microbiology, Faculty of Biological Sciences, Complutense University of Madrid, C/Jose Antonio Nováis, Nº12, 28040 Madrid, Spain

Correction to: *Scientific Reports* 10.1038/s41598-025-13194-x, published online 30 July 2025

The original version of this Article contained an error in the Methods section, under the subheading ‘Drugs and their administration’.

In the original version of this Article, the sequence GTSFLSPAQKPQGRRPPRM (specific from goldfish) was incorrect due to a transcription error. This sequence corresponds to a region of ghrelin from Carassius auratus, according to the published preproghrelin sequence available in GenBank (accession number AF454389.1).

As a result, the sentence

“The ghrelin used in this study was synthesized by Bachem (Bubendorf, Switzerland) upon request, based on the goldfish ghrelin sequence: GSSFLSPYAGKKPPKPSQP, a 19-amino acid peptide acylated with an octanoyl group at the serine-3 position (GHR-S3).”

now reads:

“The ghrelin used in this study was synthesized by Bachem (Bubendorf, Switzerland) upon request, based on the goldfish ghrelin sequence: GTSFLSPAQKPQGRRPPRM, a 19-amino acid peptide acylated with an octanoyl group at the serine-3 position (GHR-S3).”

The original Article has been corrected.

