# Supplementary material for: Food reward entrainment increases mealtime anxiety in goldfish via a ghrelin-dependent mechanism
Source: Sci Rep. 2025 Jul 30;15:27768. doi: 10.1038/s41598-025-13194-x (PMC12311043; doi:10.1038/s41598-025-13194-x)
Supplement: Supplementary file 1 — Supplementary Material 1 [file 41598_2025_13194_MOESM1_ESM.docx]

**
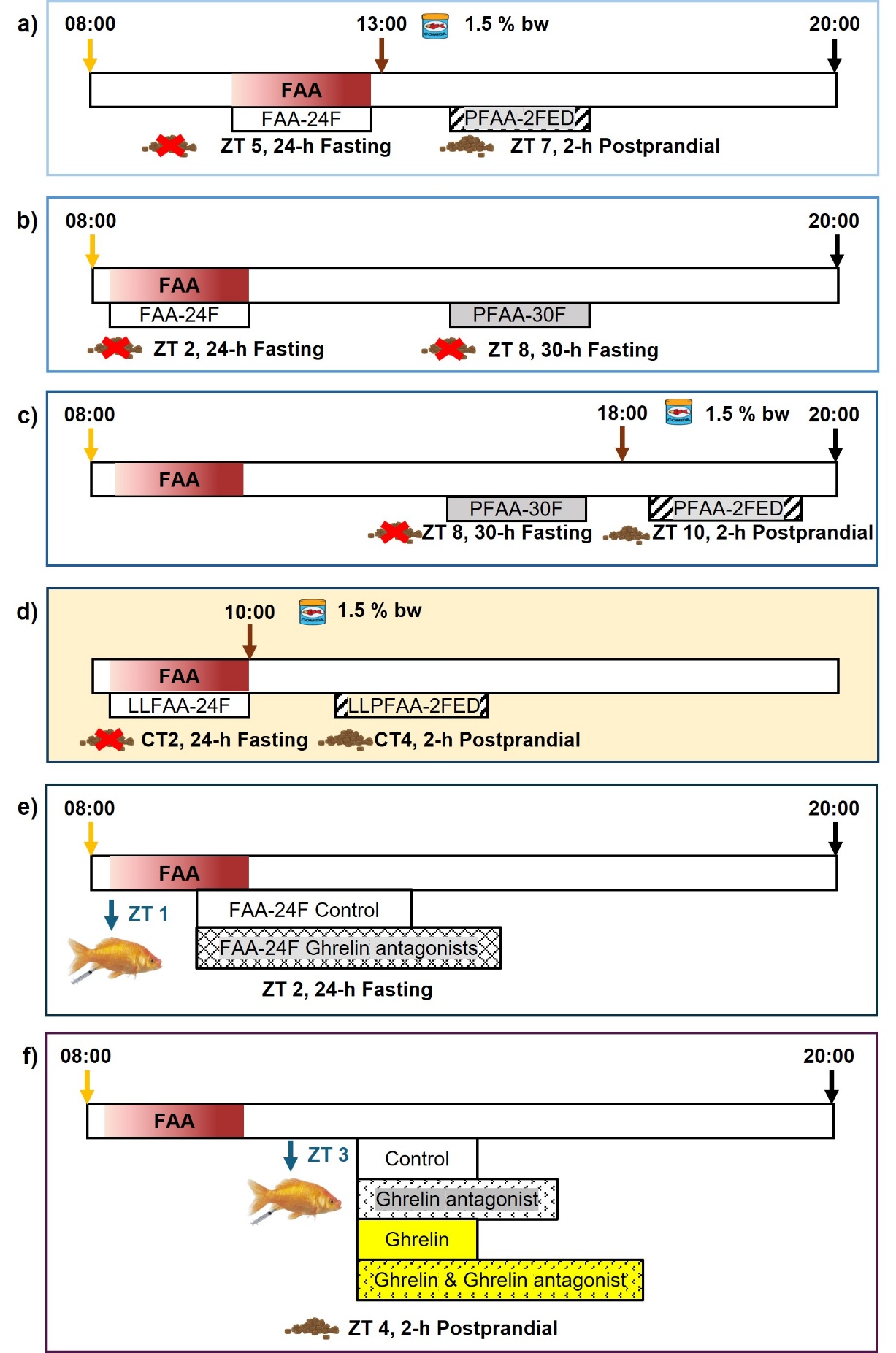
**

**Figure S1. Experimental designs to measure anxiety-like behavior under different feeding conditions and at different times of day.** For more details, see the text. FAA, food anticipatory activity period; PFAA, post food anticipatory activity period; ZT, *Zeitgeber* Time at which the behavioral tests were performed under 12L:12D photoperiod (Fig, a-c, e); CT, *Circadian* Time at which the behavioral tests were performed under LL conditions (Fig. d); ZT is blue when IP injection time (Fig. e, f); Red box, FAA period; Grey box, post FAA period and fasted animals; White stripped box, post FAA period and fed animals; Brown arrow, feeding time; LL and yellow background, animals acclimated in constant light; Blue arrow: time of the intraperitoneal injection.


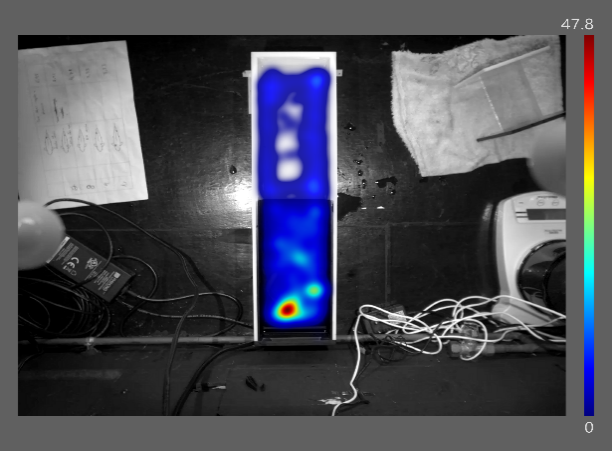

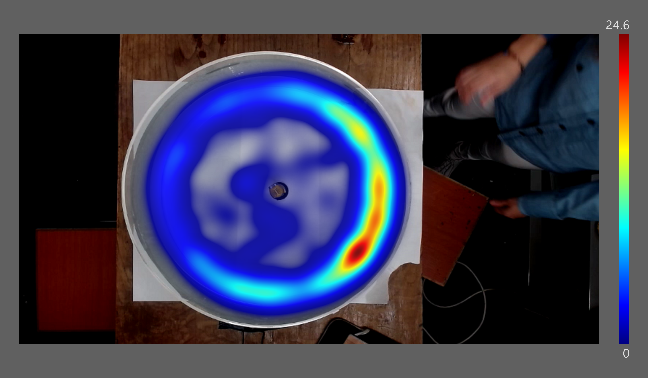

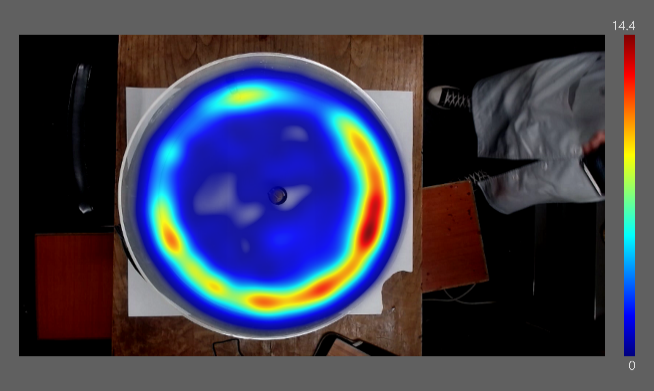

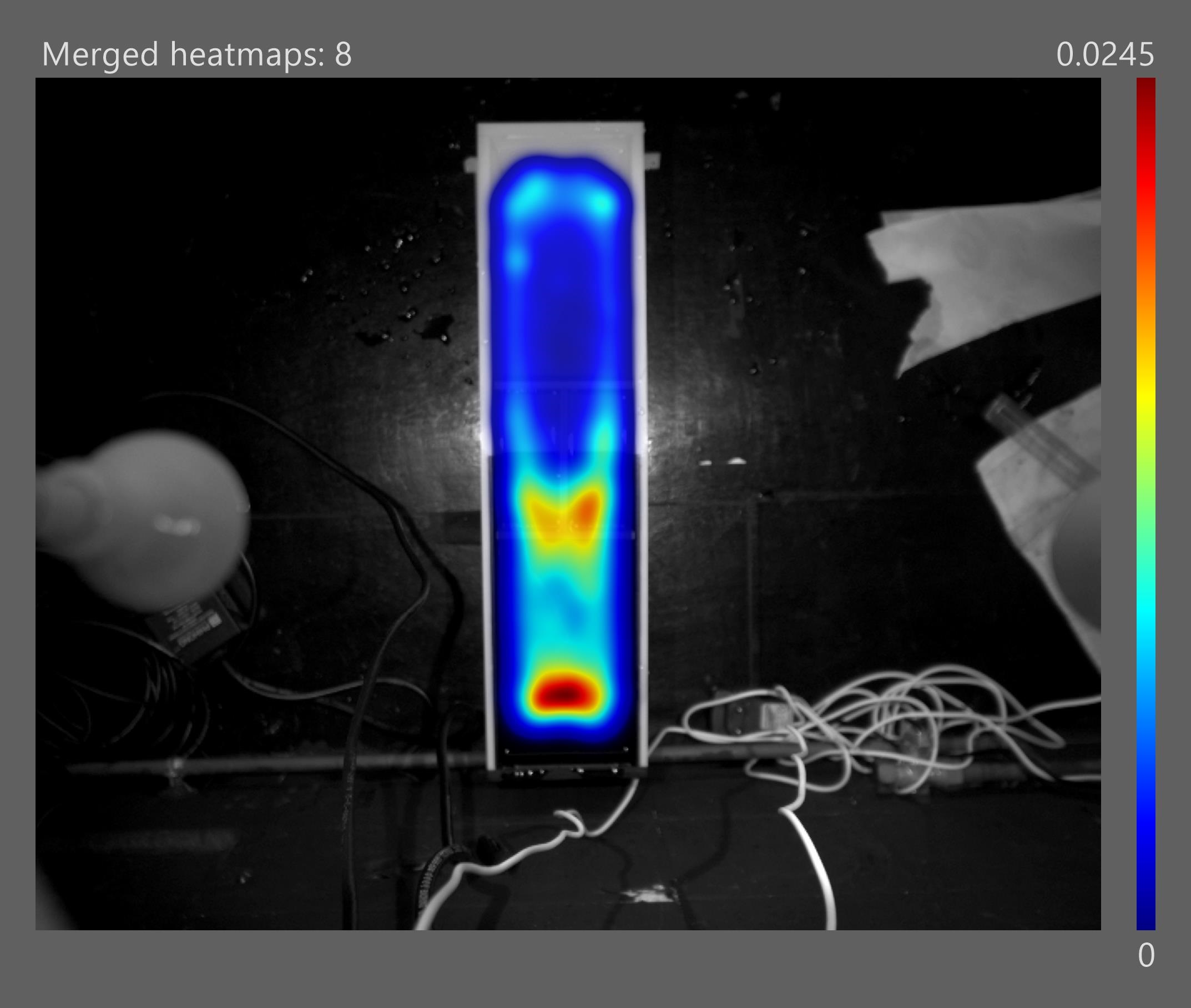


**Low**

**High**


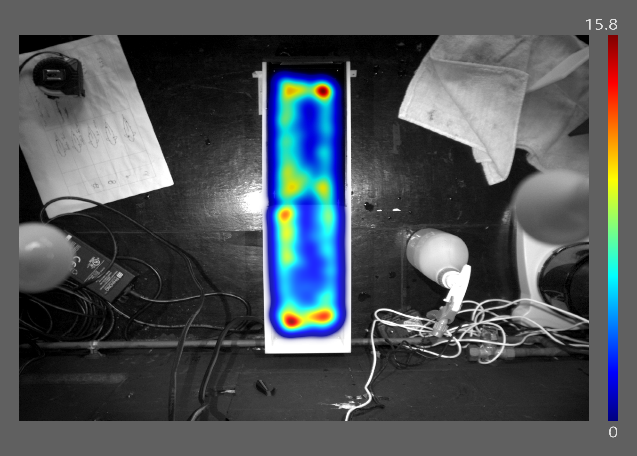


**PFAA-2FED**

**FAA-24F**

**b)**

**d)**

**c)**

**a)**

**Figure S2. Heatmaps of the open field test (a–b) and the black-white preference (c-d; black side at left) tests for fish during their FAA (a, c) or post-FAA at 2 h postprandial (b, d).** Heatmaps generated by automated video tracking (Ethovision, Noldus), illustrate the time fish stay in each part of the arena (mean of n=20 fish/group) from blue (less time) to red (higher time). FAA, food anticipatory activity; PFAA, post-food anticipatory activity; 24F, 24-h of fasting.


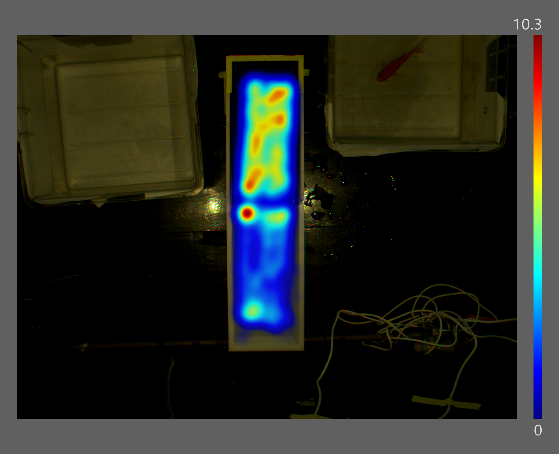

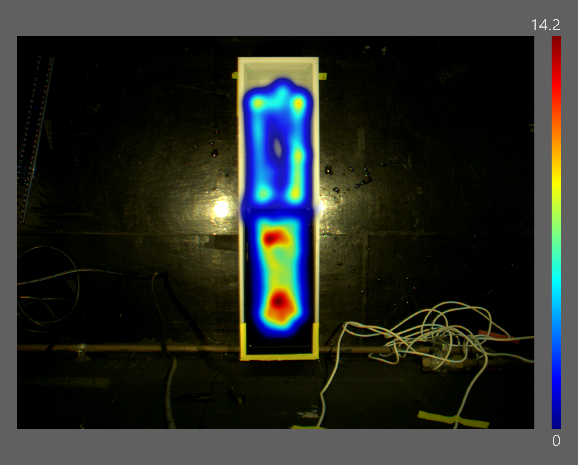

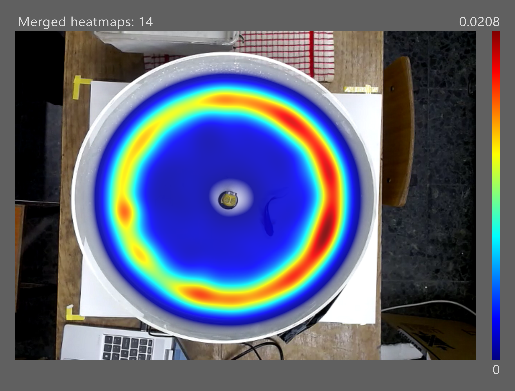

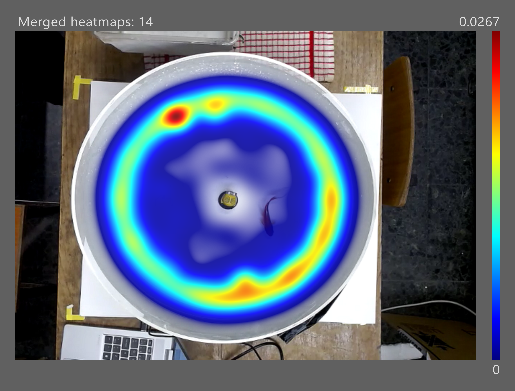

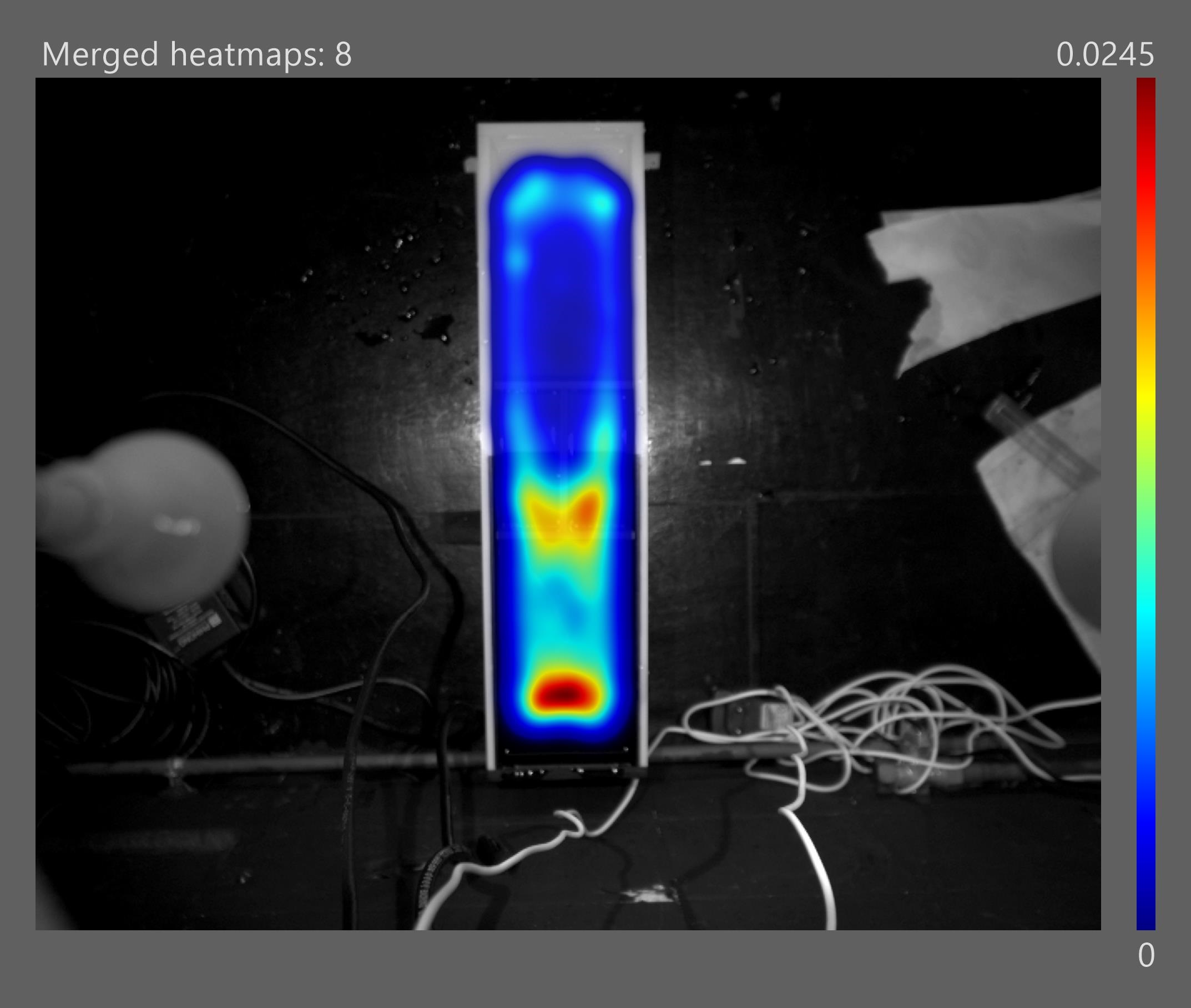


**Low**

**High**

**FAA-24F**

**PFAA-30F**

**b)**

**d)**

**c)**

**a)**

**Figure S3. Heatmaps of the open field test (a–b) and the black-white preference (c-d; black side at left) tests for fish in the FAA and 24-h of fasting (a, c) or in the post-FAA period after 30-h fasting (b, d).** Heatmaps generated by automated video tracking (Ethovision, Noldus), illustrate the time fish stay in each part of the arena (mean of n=14 fish/group) from blue (less time) to red (higher time). FAA, food anticipatory activity; PFAA, post-food anticipatory activity; 24F, 24-h of fasting; 30F 30-h of fasting.


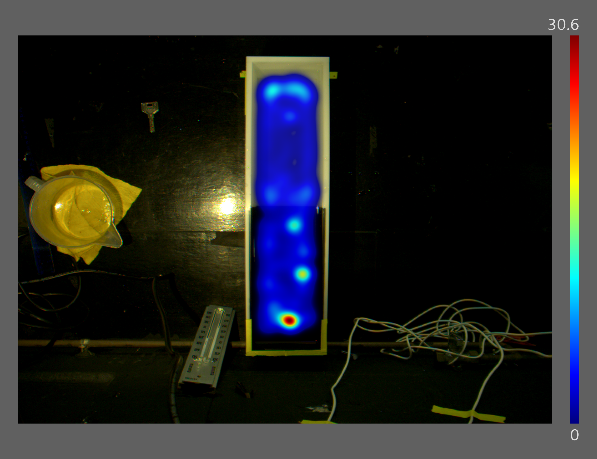

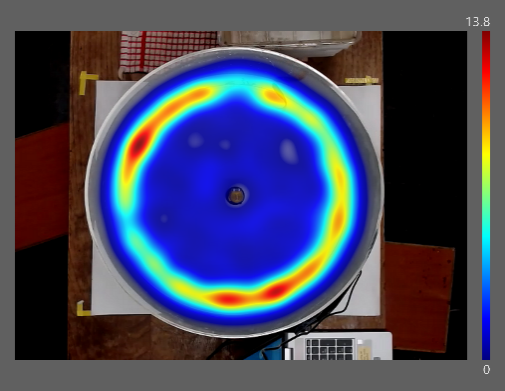

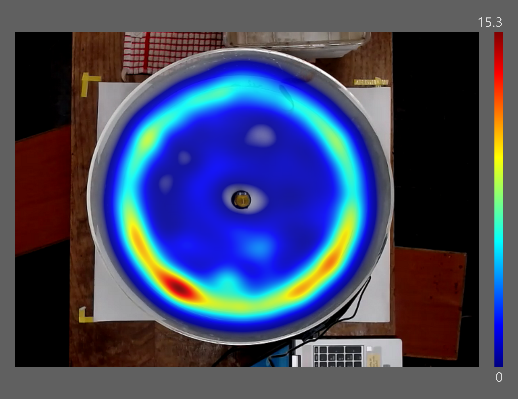

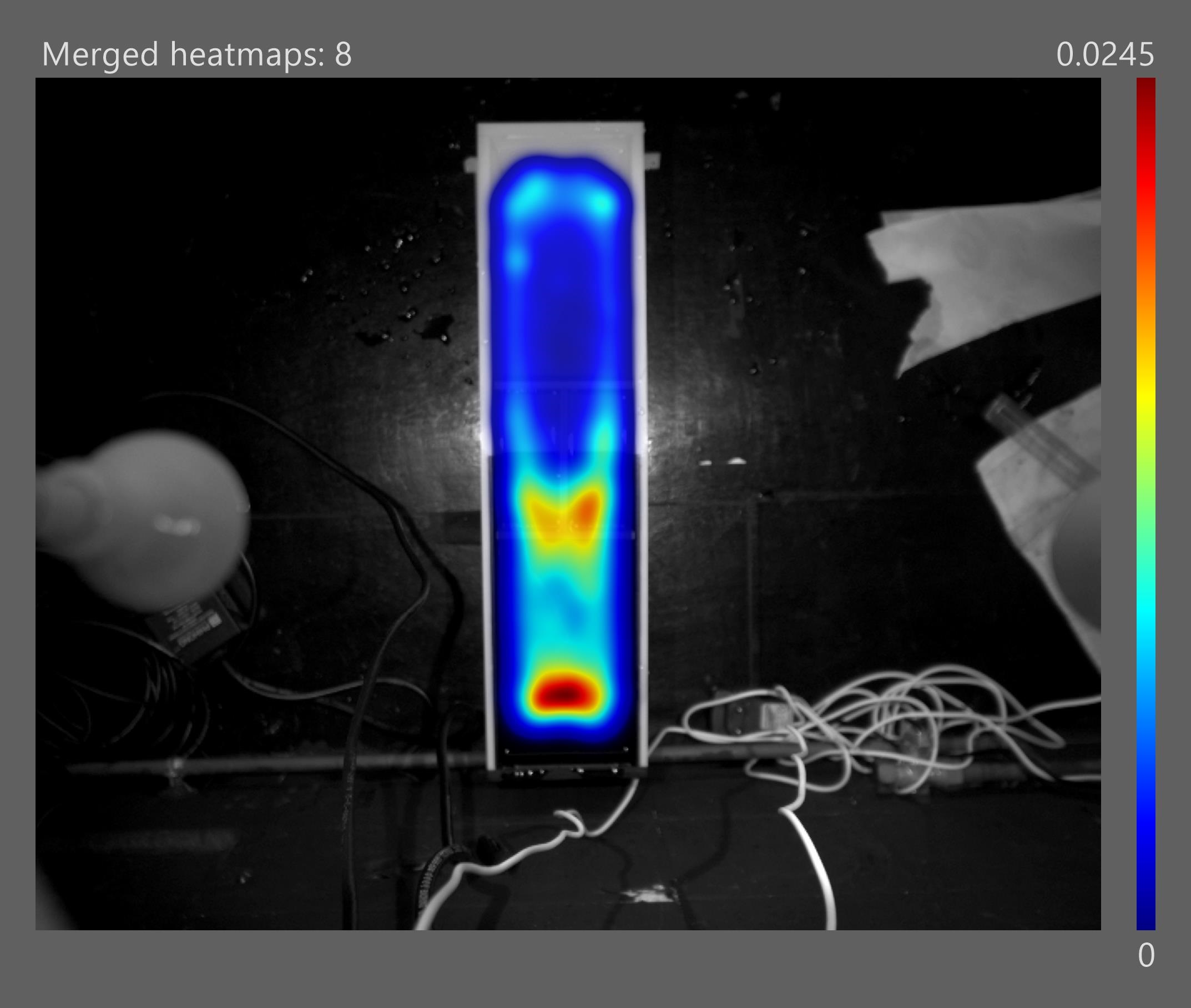


**Low**

**High**


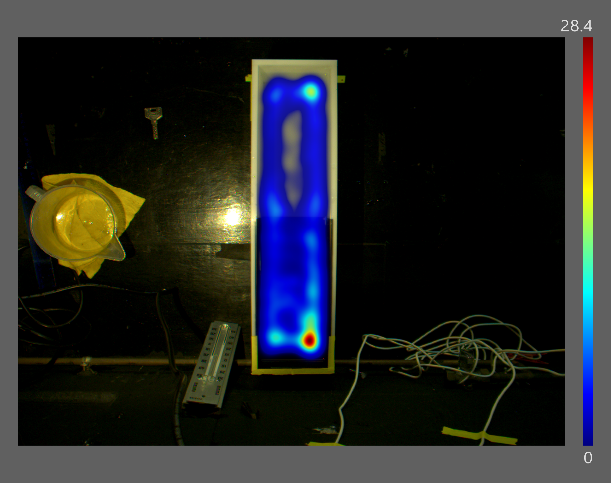


**PFAA-30F**

**PFAA-2FED**

**b)**

**d)**

**c)**

**a)**

**Figure S4. Heatmaps of the open field test (a–b) and the black-white preference (c-d; black side at left) tests for fish after the FAA period with 30-h of fasting (a, c) or at 2 hours post-prandial (b, d).** Heatmaps generated by automated video tracking (Ethovision, Noldus), illustrate the time fish stay in each part of the arena (mean of n=16 fish/group) from blue (less time) to red (higher time). PFAA, post-food anticipatory activity; 30F, 30-h fasting.

**LLPFAA-2FED**


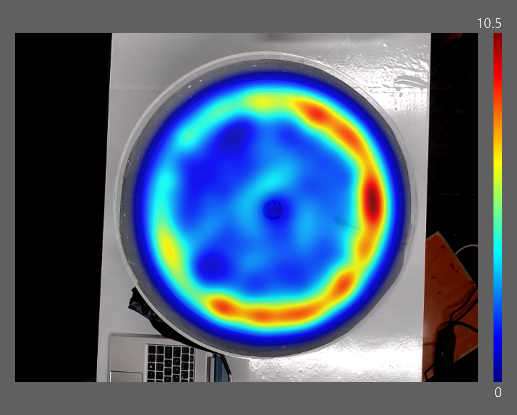

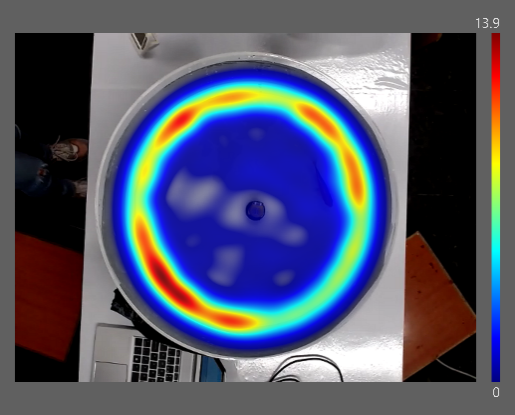

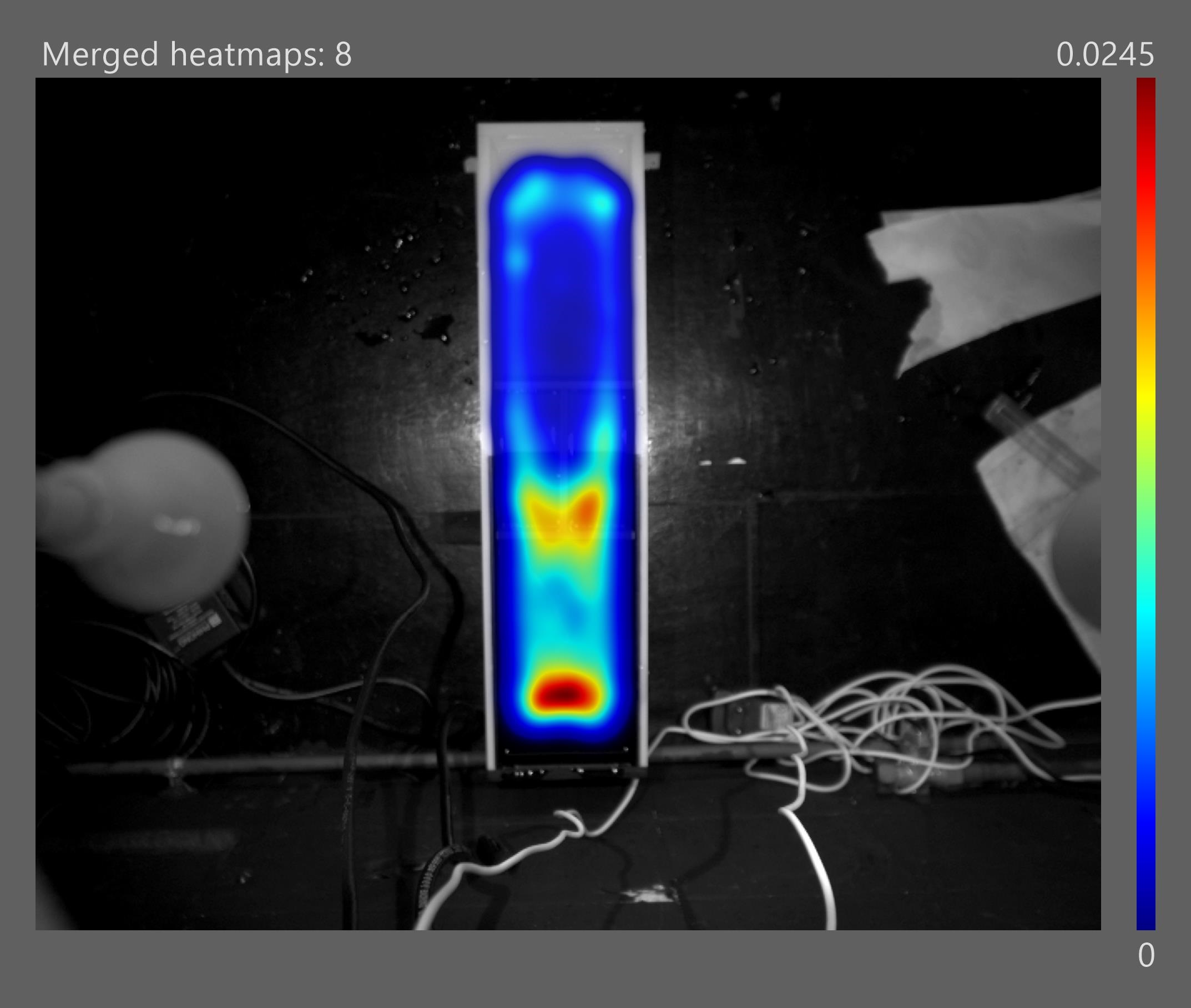


**Low**

**High**


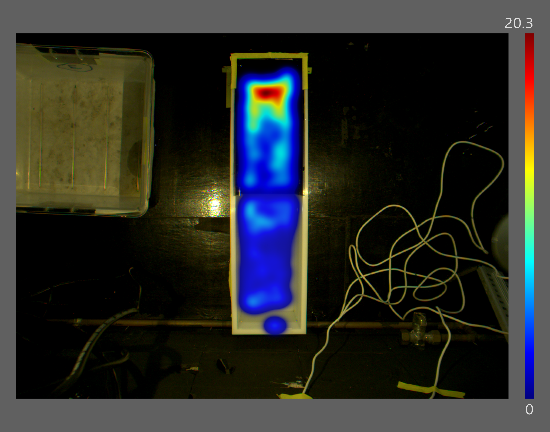

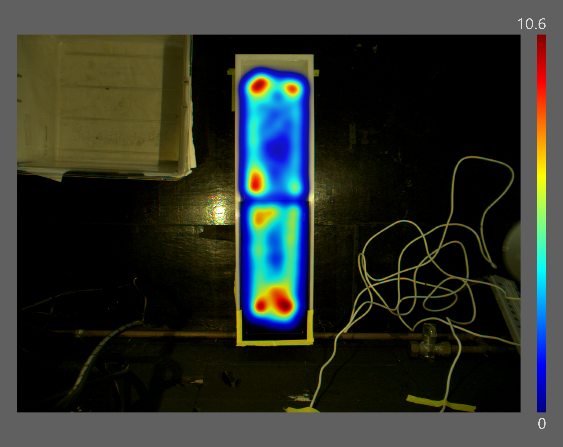


**LLFAA-24F**

**b)**

**d)**

**c)**

**a)**

**Figure S5. Heatmaps of the open field test (a–b) and the black-white preference (c-d, black side at left) tests for fish in the FAA period and 24-h fasting (a, c) or in the post-FAA period at 2 hours post-prandial (b, d) in animals under constant light.** Heatmaps generated by automated video tracking (Ethovision, Noldus), illustrate the time fish stay in each part of the arena (mean of n=14 fish/group) from blue (less time) to red (higher time). FAA, food anticipatory activity; LL, constant light; PFAA, post-food anticipatory activity; 24F, 24-h of fasting.

**D-LYS**


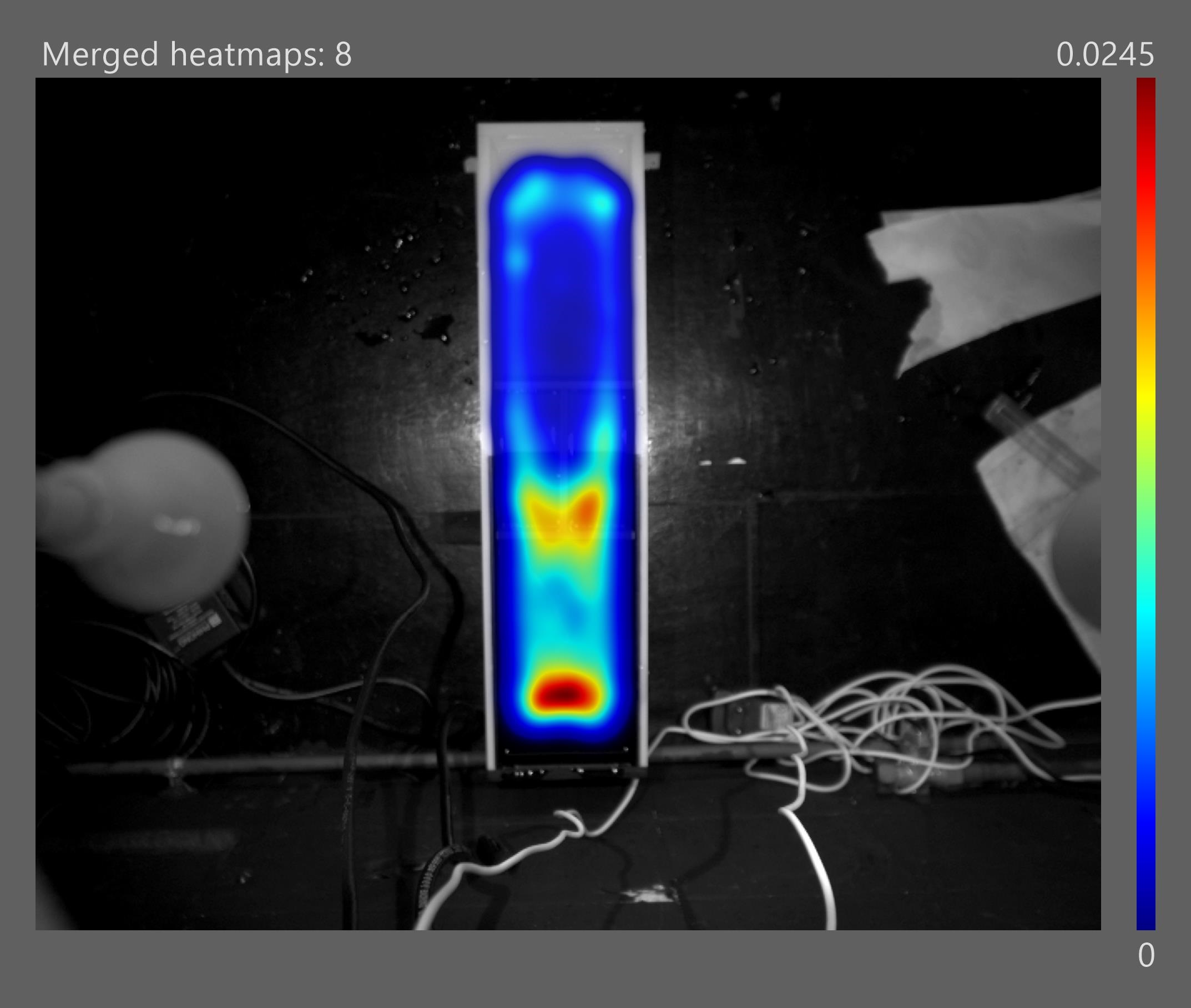


**Low**

**High**

**JMV2950**

**CONTROL**


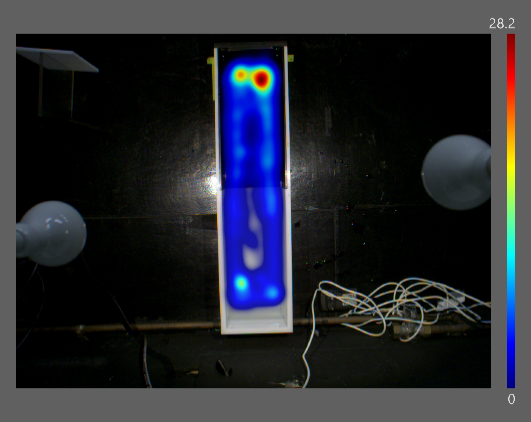

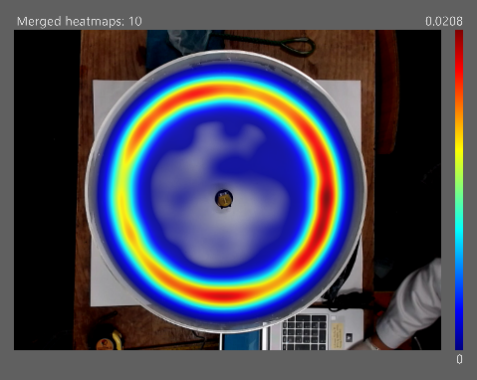

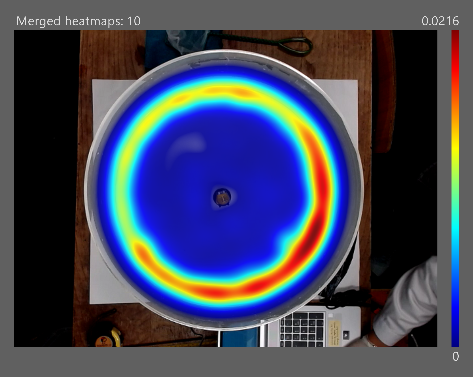

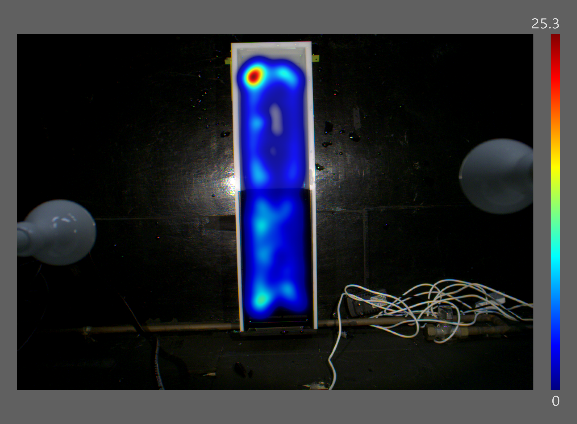


**b)**

**e)**

**d)**

**a)**


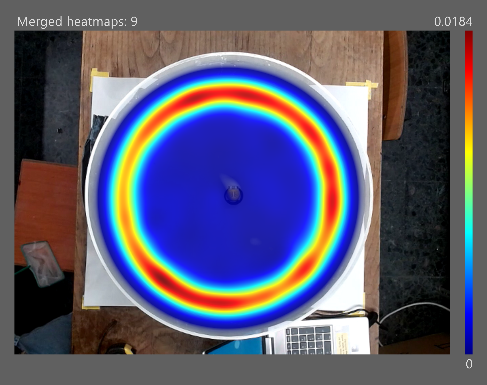

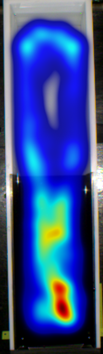


**c)**

**f)**

**Figure S6: Heatmaps of the open field test (a–c) and the black/white preference (d-f) tests for fish in the FAA period and 24-h fasting treated with teleost saline (a, d) or with the ghrelin antagonists: JMV2959 (b, e) or D-lys (c, f ) during the FAA.** Heatmaps generated by automated video tracking (Ethovision, Noldus), illustrate the time fish stay in each part of the arena (mean of n=9 fish/group) from blue (less time) to red (higher time).

*****

**Fig. S7**. **Effect of feed intake of Ghrelin for 2 hours at 2-h postprandial.** Mean values + S.E.M are represented (n=10 fish/group).


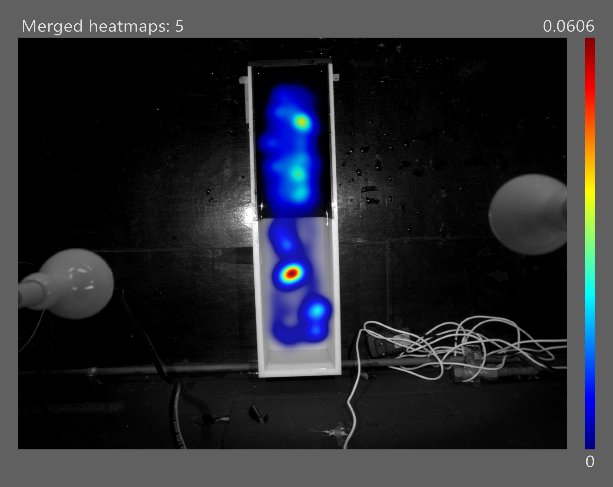

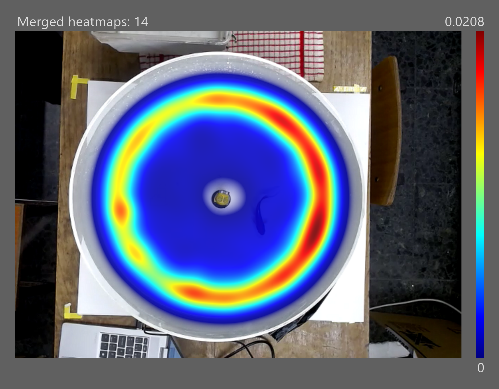


**h)**

**d)**


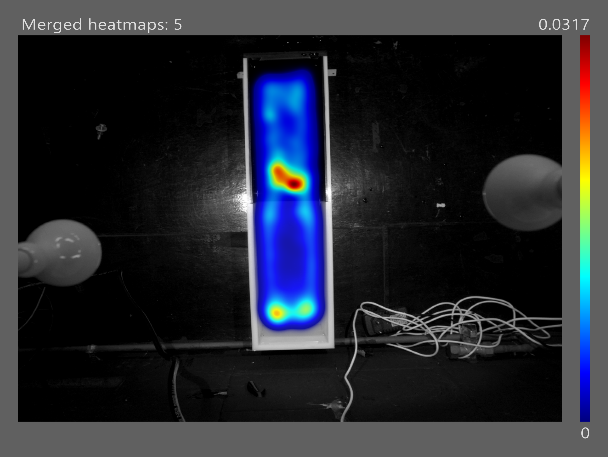

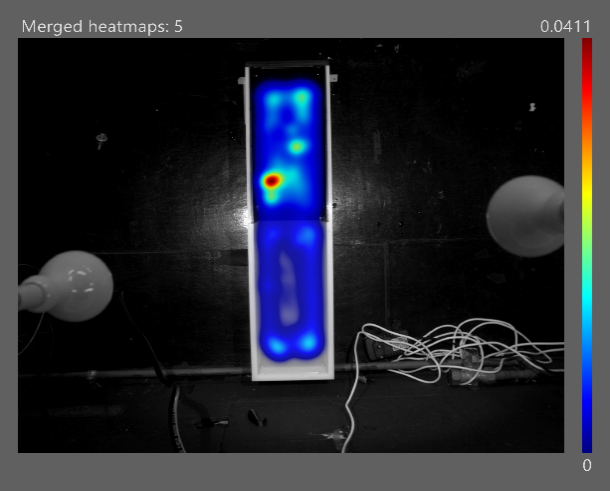

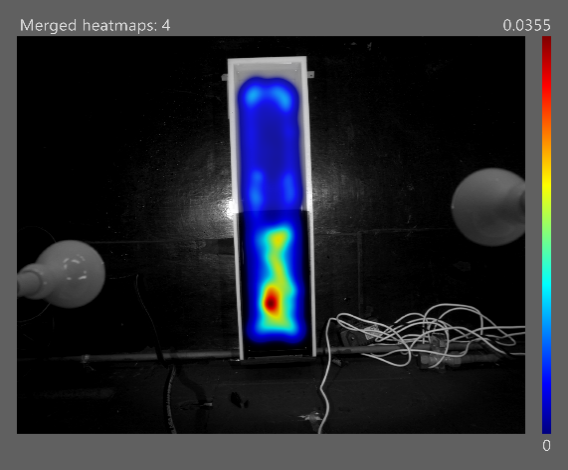

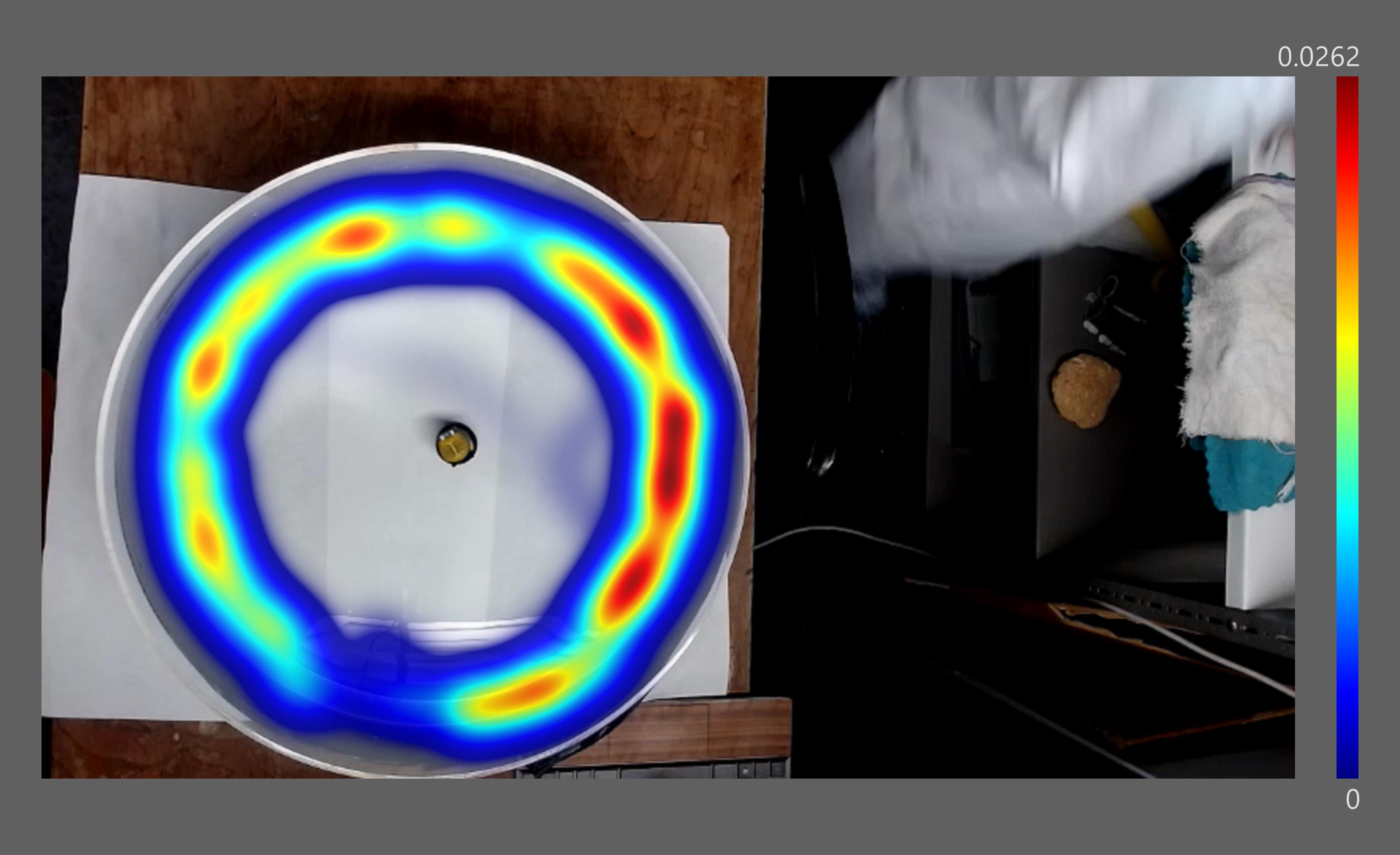

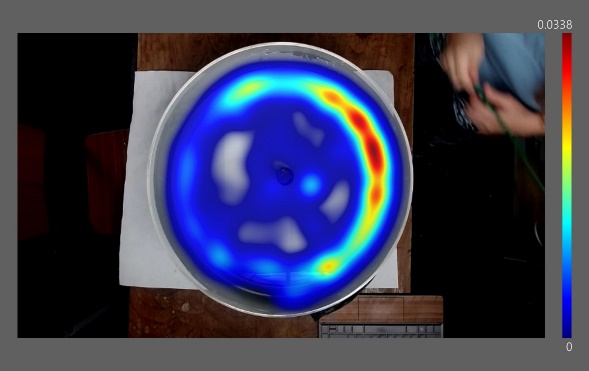

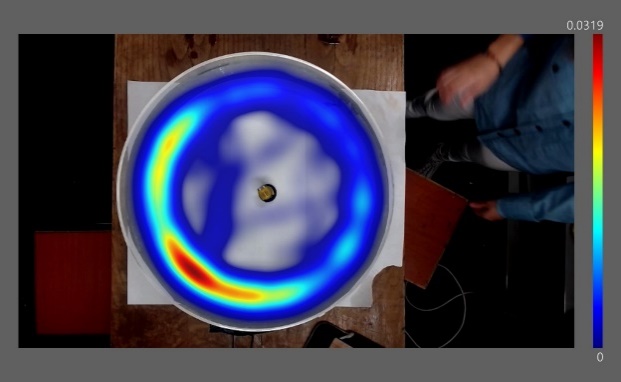


**D-LYS**


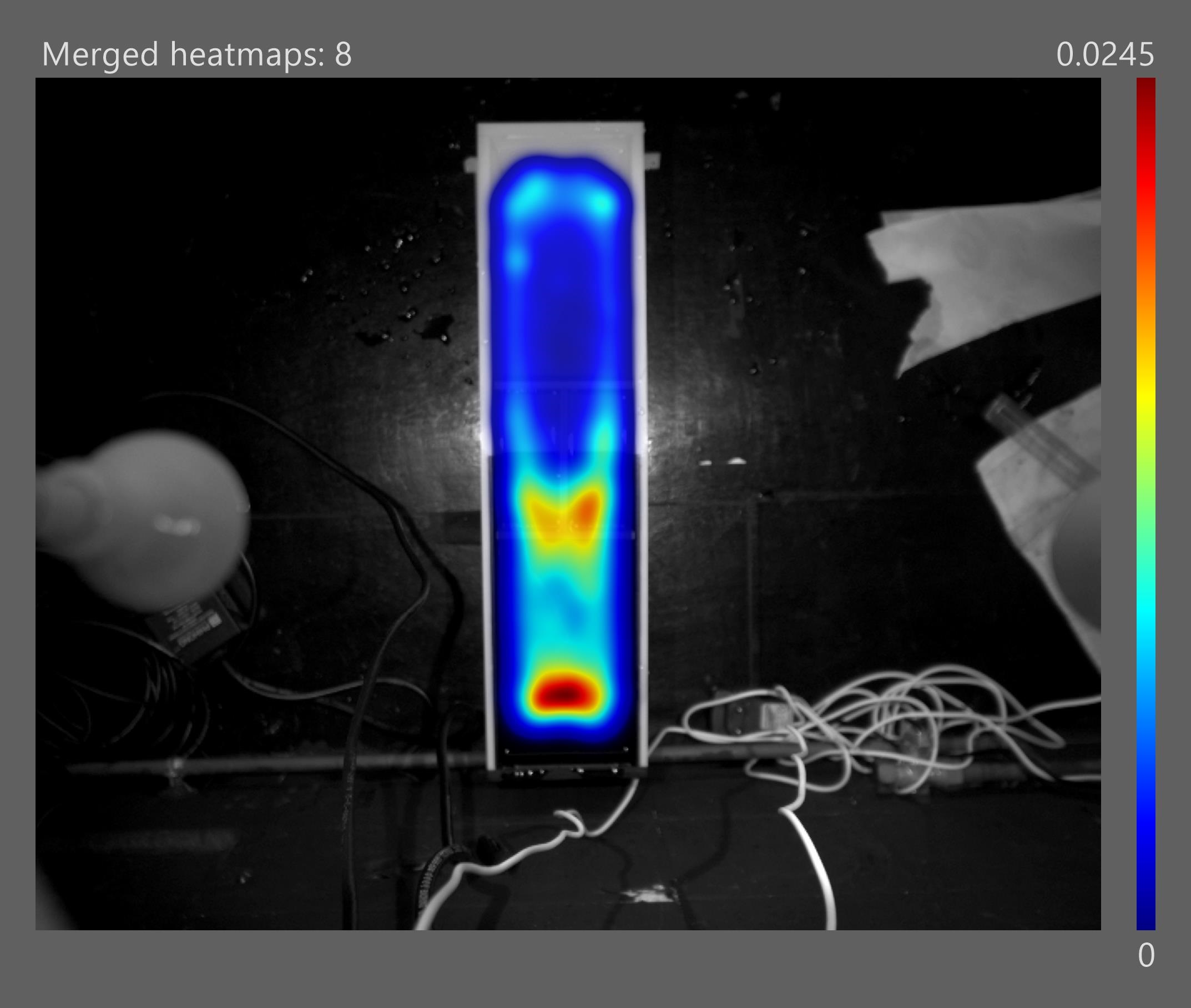


**Low**

**High**

**CONTROL**

**b)**

**f)**

**e)**

**a)**

**c)**

**g)**

**GHRELINA**

**D + G**

**Fig. S8.** **Heatmaps of the open field test (a–d) and the black/white preference (e-h) tests for fish at 2-h postpandrial treated with teleost saline (a, e) or with the ghrelin antagonist D-lys (b, f) or ghrelin alone (c, g) or Dlys and Ghrelin (d, g).** Heatmaps generated by automated video tracking (Ethovision, Noldus), illustrate the time fish stay in each part of the arena (mean of n=8-10 fish/group) from blue (less time) to red (higher time). D+G: D-lys + Ghrelin.

**Table S1. Statistical parameters for each behavioral variable analyzed for fish in the FAA or after the post-FAA period at 2-h postprandial.** N, total number of animals, p-value, probability of null hypothesis in the Student T-test (significant differences are represented in bold), df: degrees of freedom. $ parameters transformed by log 10 o # transformed by square root.

| OPEN FIELD TEST | | | | |
| --- | --- | --- | --- | --- |
|  | N | t | p-value | df |
| % time in open field | 40 | 3.864 | **0.0004** | 38 |
| Velocity in the open zone (cm/s) $ | 40 | 2.631 | **0.012** | 38 |
| No of entries to open $ | 40 | -4.474 | **0.00007** | 38 |
| Latency to open field (s) # | 40 | 3.042 | **0.004** | 38 |
| No of entries to the center # | 40 | -2.897 | **0.006** | 38 |
| Latency to the center (s) | 40 | 1.749 | 0.0921 | 38 |
| BLACK-WHITE TEST | | | | |
| % time in the white zone | 40 | -3.442 | **0.001** | 38 |
| No of entries to white zone | 40 | -1.951 | 0.059 | 38 |
| Latency to white zone (s) $ | 40 | 2.824 | **0.011** | 38 |

**Table S2. Statistical parameters for each behavioral variable analyzed for fish in the FAA and 24-h of fasting or in the post-FAA period after 30-h fasting.** N, total number of animals, p-value, probability of null hypothesis in the Student T-test (significant differences are represented in bold), df: degrees of freedom. $ parameters transformed by log 10 o # transformed by square root.

| OPEN FIELD TEST | | | | |
| --- | --- | --- | --- | --- |
|  | N | t | p-value | df |
| % time in open field | 28 | -2.477 | **0.020** | 26 |
| Velocity in the open zone (cm/s) $ | 28 | 0.889 | 0.382 | 26 |
| No of entries to open $ | 28 | 2.064 | **0.049** | 26 |
| Latency to open field (s) # | 28 | 1.013 | 0.320 | 26 |
| No of entries to the center # | 28 | -2.185 | **0.038** | 26 |
| Latency to the center (s) # | 28 | 1.170 | 0.253 | 26 |
| BLACK-WHITE TEST | | | | |
| % time in the white zone | 28 | 3.988 | **0,0005** | 26 |
| No entries to white zone $ | 28 | -3.491 | **0,002** | 26 |
| Latency to white zone (s) # | 28 | 0.536 | 0,596 | 26 |

**Table S3. Statistical parameters for each behavioral variable analyzed for fish after the FAA period with 30-h of fasting or at 2 hours post-prandial.** N, total number of animals, p-value, probability of null hypothesis in the Student T-test, df: degrees of freedom. $ parameters transformed by log 10 o # transformed by square root.

| OPEN FIELD TEST | | | | |
| --- | --- | --- | --- | --- |
|  | N | t | p-value | df |
| % time in open field $ | 31 | 0.524 | 0.604 | 29 |
| Velocity in the open zone (cm/s) | 31 | 0.429 | 0.671 | 29 |
| No of entries to open | 31 | 0.755 | 0.456 | 29 |
| Latency to open field (s) # | 31 | 1.037 | 0.308 | 29 |
| No of entries to the center # | 31 | 1.162 | 0.255 | 29 |
| Latency to the center (s) $ | 31 | 0.089 | 0.930 | 29 |
| BLACK-WHITE TEST | | | | |
| % time in the white zone | 31 | 0.613 | 0.545 | 29 |
| No entries to white zone | 31 | -1.207 | 0.237 | 29 |
| Latency to white zone (s) $ | 31 | 0.429 | 0.671 | 29 |

**Table S4. Statistical parameters for each behavioral variable analyzed tests for fish in the FAA period and 24-h fasting or in the post-FAA period at 2 hours post-prandial and constant light.** N, total number of animals, p-value, probability of null hypothesis in the Student T-test (significant differences are represented in bold), df: degrees of freedom. $ parameters transformed by log 10 o # transformed by square root.

| OPEN FIELD TEST | | | | |  |
| --- | --- | --- | --- | --- | --- |
|  | N | t | p-value | df | |
| % time in open field | 27 | -2.180 | **0.039** | 25 | |
| Velocity in the open zone (cm/s) $ | 27 | 2.176 | **0.039** | 25 | |
| No of entries to open | 27 | -1.759 | 0.091 | 25 | |
| Latency to open field (s) # | 27 | 1.675 | 0.106 | 25 | |
| Nº of entries to the center # | 27 | -0.779 | 0.443 | 25 | |
| Latency to the center (s) $ | 27 | 1.432 | 0.165 | 25 | |
| BLACK-WHITE TEST | | | | |  |
| % time in the white zone | 27 | 2.252 | **0.033** | 25 | |
| No entries to white zone | 27 | -1.364 | 0.185 | 25 | |
| Latency to white zone (s) # | 27 | 2.291 | **0.031** | 25 | |

**Table S5. Statistical parameters for each behavioral variable analyzed for fish treated with the ghrelin antagonist, JMV2959, or teleost saline.** N, total number of animals, p-value, probability of null hypothesis in the Student T-test (significant differences are represented in bold), df: degrees of freedom. $ parameters transformed by log 10 o # transformed by square root.

| OPEN FIELD TEST | | | | |
| --- | --- | --- | --- | --- |
|  | N | t | p-value | df |
| % time in open field $ | 18 | 2.859 | **0.011** | 16 |
| Velocity in the open zone (cm/s) | 18 | -2.573 | **0.020** | 16 |
| No of entries to open | 18 | 2.083 | 0.054 | 16 |
| Latency to open field (s) | 18 | -1.076 | 0.298 | 16 |
| No of entries to the center # | 18 | 2.513 | **0.023** | 16 |
| Latency to the center (s) $ | 18 | -2.277 | **0.037** | 16 |
| BLACK-WHITE TEST | | | | |
| % time in the white zone | 18 | 0.571 | 0.576 | 16 |
| No entries to white zone | 18 | -1.615 | 0.126 | 16 |
| Latency to white zone (s) # | 18 | -2.927 | **0.009** | 16 |

**Table S6. Statistical parameters for each behavioral variable analyzed for fish treated with the ghrelin antagonist, D-lys, or teleost saline.** N, total number of animals, p-value, probability of null hypothesis in the Student T-test (significant differences are represented in bold), df: degrees of freedom. $ parameters transformed by log 10 o # transformed by square root.

| OPEN FIELD TEST | | | | |
| --- | --- | --- | --- | --- |
|  | N | t | p-value | df |
| % time in open field $ | 18 | 2.875 | **0.006** | 16 |
| Velocity in the open zone (cm/s) | 18 | -1.488 | 0.08 | 16 |
| No of entries to open $ | 18 | 1.392 | 0.092 | 16 |
| Latency to open field (s) # | 18 | -2.267 | **0.047** | 16 |
| No of entries to the center | 18 | 1.204 | 0.124 | 16 |
| Latency to the center (s) $ | 18 | -2.082 | 0.055 | 16 |
| BLACK-WHITE TEST | | | | |
| % time in the white zone $ | 18 | -1.991 | **0.032** | 16 |
| No entries to white zone # | 18 | -1.301 | 0.106 | 16 |
| Latency to white zone (s) | 18 | -0.251 | 0.402 | 16 |

**Table S7. Statistical parameters for each behavioral variable analyzed for fish treated with the combination of Ghrelin and its antagonist D-lys.** N, total number of animals, p-value, probability of null hypothesis in the Student T-test (significant differences are represented in bold), df: degrees of freedom. $ parameters transformed by log 10 o # transformed by square root. G: factor ghrelin, D: factor D-lys and G·D: interaction Ghrelin and D-lys.

| OPEN FIELD TEST | | | | | | | | |
| --- | --- | --- | --- | --- | --- | --- | --- | --- |
|  | | G | | D | | G·D | |  |
|  | N | p-valor | F | p-valor | F | p-valor | F | df |
| % time in open field $ | 36 | **< 0.001** | 20.650 | **0.001** | 12.268 | 0.507 | 0.239 | 35 |
| Velocity in the open zone (cm/s) # | 36  36 | 0.097 | 2.961 | **0.029** | 5.235 | **0.04** | 4.61 | 35 |
|  |  | One way ANOVA: p: **0.013**; F: 4.221 | | | | | | 35 |
| No of entries to open # | 36 | **0.018** | 6.272 | 0.260 | 1.316 | 0.166 | 2.009 | 35 |
| Latency to open field (s) # | 36 | **0.016** | 6.530 | **0.002** | 11.240 | 0.095 | 2.973 | 35 |
| Nº of entries to the center # | 36 | **0.041** | 4.678 | **0.043** | 4.628 | 0.827 | 0.049 | 35 |
| Latency to the center (s) | 36 | **0.025** | 5.564 | 0.287 | 1.173 | 0.602 | 0.277 | 35 |
| BLACK-WHITE TEST | | | | | | | |  |
| % time in the white zone | 36 | 0.450 | 0.589 | 0.086 | 3.188 | 0.073 | 3.509 | 35 |
| No entries to white zone | 36 | 0.174 | 1.960 | **< 0.001** | 15.741 | **0.043** | 4.721 | 35 |
|  |  | One way ANOVA: **p: 0.002**; F: 6.746 | | | | | | 35 |
| Latency to white zone (s) # | 36 | **0.016** | 6.607 | 0.127 | 2.036 | **0.044** | 4.609 | 35 |
|  |  | One way ANOVA: **p: 0.028**; F: 3.586 | | | | | | 35 |
